# Protein Sequencing with Single Amino Acid Resolution Discerns Peptides That Discriminate Tropomyosin Proteoforms

**DOI:** 10.1021/acs.jproteome.4c00978

**Published:** 2025-06-10

**Authors:** Natchanon Sittipongpittaya, Kenneth A. Skinner, Erin D. Jeffery, Emily F. Watts, Gloria M. Sheynkman

**Affiliations:** † Department of Molecular Physiology and Biological Physics, 12349 University of Virginia, Charlottesville, Virginia 22903, United States; ‡ Department of Biochemistry and Molecular Genetics, University of Virginia, Charlottesville, Virginia 22903, United States; § UVA Comprehensive Cancer Center, University of Virginia, Charlottesville, Virginia 22903, United States; ∥ 497903 Quantum-Si Incorporated, 29 Business Park Drive, Branford, Connecticut 06405, United States

**Keywords:** amino acid variant, single-molecule peptide sequencing, tropomyosin, genetic variants, alternative splicing, phosphorylation, recognizer-based sequencing, proteoforms, isobaric peptides, proteogenomics

## Abstract

Protein variants of the same gene—proteoforms—can have high molecular similarity yet exhibit different biological functions. Thus, the identification of unique peptides that unambiguously map to proteoforms can provide crucial biological insights. In humans, four human tropomyosin (TPM) genes produce similar proteoforms that can be challenging to distinguish with standard proteomics tools. For example, TPM1 and TPM2 share 85% sequence identity with amino acid substitutions that play unique roles in muscle contraction and myopathies. In this study, we evaluated the ability of the recently released Platinum single-molecule protein sequencer to detect proteoform-informative peptides. Platinum employs fluorophore-labeled recognizers that reversibly bind to cognate N-terminal amino acids (NAAs), enabling polypeptide sequencing within nanoscale apertures of a semiconductor chip that can accommodate single peptide molecules. As a proof of concept, we evaluated the ability of Platinum to distinguish three main types of proteoform variation: paralogue-level, transcript-level, and post-translational modification (PTM). We distinguished paralogous TPM1 and TPM2 peptides differing in a single isobaric residue (leucine/isoleucine). We also distinguished tissue-specific TPM2 spliceforms. Notably, we found that a phosphotyrosine-modified peptide displayed a reduced recognizer affinity for tyrosine, showing sensitivity to PTMs. This study paves the way for the targeted detection of proteoform biomarkers at the single molecule level.

## Graphical abstract

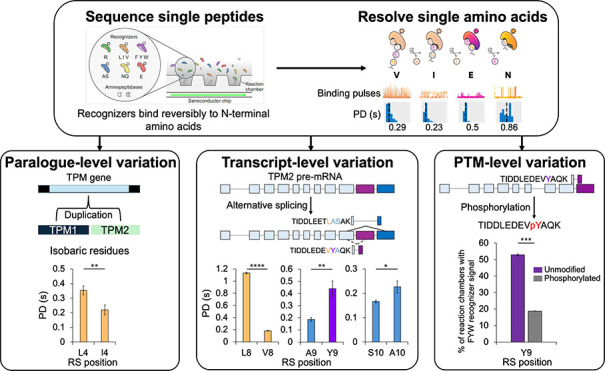

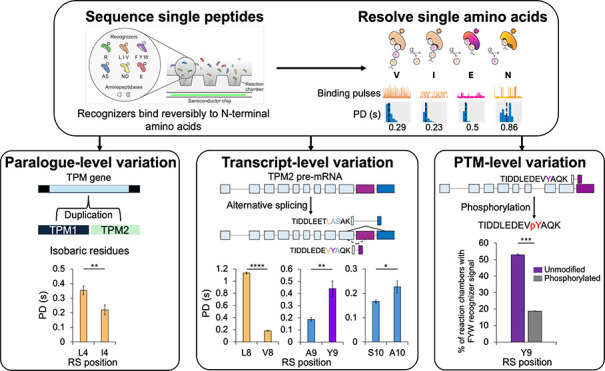

## Introduction

Proteins serve as the vital molecular bridge between genotype and phenotype, making their functional characterization critical for complete understanding of biological processes.^1^ Protein function, however, is not fully encoded in the genome. Alternative splicing (AS) of pre-mRNA transcripts can produce distinct protein isoforms.^2,3^ Genes can also be regulated at the post-transcriptional level, and proteins often undergo post-translational modifications (PTMs) that can alter function.^4^ The protein products of genetic, post-transcriptional, and post-translational variation—collectively termed proteoforms^5^—can exhibit strikingly diverse functions that give rise to phenotypes in human health and disease.^2,4^ Thus, detecting these proteoforms is key to understanding the molecular link between protein function and phenotype.

Characterizing proteoform expression can present challenges. Currently, the existence of most proteoforms is inferred from mRNA transcripts^6^ and putative PTM sites.^7^ However, not all mRNA transcripts are translated into protein isoforms,^8^ and some transcripts are degraded without translation.^9–11^ In addition, even if putative PTM sites for all proteoforms are known, the exact proteoforms in a sample cannot be identified unless PTMs are detected and localized to specific sites.^12^ Indeed, the vast majority of predicted proteoforms have yet to be validated at the peptide or protein level.^13,14^ To bridge this gap, it is necessary to leverage a range of analytical technologies with varying capabilities both independently and in combination.

Traditional antibody-based assays cannot distinguish proteoforms unless antibodies with high proteoform specificity are available.^15,16^ While mass spectrometry (MS)-based methods have benefits such as high throughput and versatility, they can miss proteoform-informative peptides due to data-dependent acquisition (DDA) selection bias toward the most intense peptide ions.^17^ In addition, resolving peptides with similar physicochemical properties is challenging with size- and charge-based chromatographic methods.^18^ Recently, there has been considerable progress in identifying peptides that uniquely indicate specific proteoforms,^19,20^ an important advancement for discerning proteoforms with high amino acid sequence identity. However, to fully characterize proteoforms, it will be necessary to employ new and orthogonal detection methods capable of addressing some of the drawbacks of the current methods.

Recently, several single-molecule protein sequencing approaches have emerged with the potential to detect subtle amino acids and variations.^21–23^ Recognizer-based sequencing, also termed next-generation protein sequencing (NGPS), was the first of these to be commercialized with the release of the Platinum instrument (Quantum-Si). Therefore, we sought to evaluate the feasibility of distinguishing proteoform-informative peptides using NGPS on Platinum. For this study, we chose a model proteoform family—tropomyosins (TPMs)—that shares high amino acid sequence similarity (Figure 1) and represents diversity at the levels of genetic polymorphisms, RNA splice variants, and PTMs.^24^

**Figure 1:**
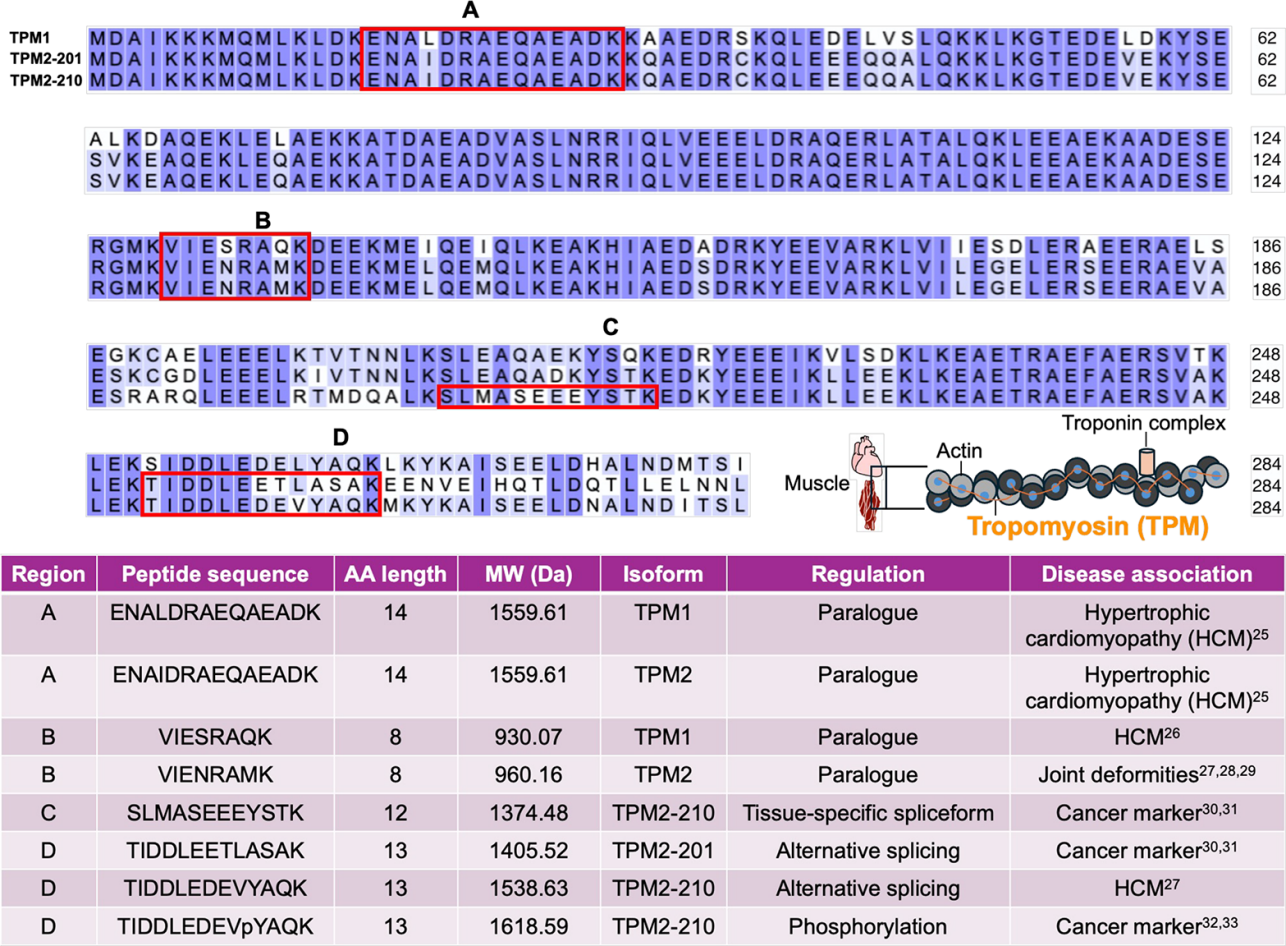
Sequence alignment of TPM1 and TPM2 isoforms and relevance of amino acid mutations to pathology. The primary structures of canonical TPM1 and two TPM2 spliceforms are shown. TPM and troponin associate with actin to form the contractile apparatus. Region A corresponds to an N-terminal region that contains isobaric peptides that differ by leucine (Leu; L) or isoleucine (Ile; I). Mutations in these paralogues have been linked with hypertrophic cardiomyopathy (HCM).^25^ Region B delineates an internal eight amino acid long region within TPM1/2 paralogues. Mutations within this region are also found in HCM^26^ and joint deformities due to the Sheldon-Hall syndrome and distal arthrogryposis.^27–29^ Region C highlights an isoform-informative peptide (SLMASEEEYSTK) that can be used as a cancer marker.^30,31^ Region D maps to an alternatively spliced C-terminal region of TPM2-201 and TPM2-210. While TIDDLEETLASAK (TPM2-201) and TIDDLEDEVYAQK (TPM2-210) share the same amino acid length, TIDDLEDEVYAQK is subject to phosphorylation on a single tyrosine residue.^32,33^

Filament proteins such as TPMs are among the most highly conserved proteins in evolution, playing critical roles in cellular architecture, cytoskeletal movement, and compartmental trafficking.^24^ TPMs are α-helical coiled coil dimers that regulate the stability of actin filaments in muscle and nonmuscle cells.^34^ Via alternative splicing, tissue-specific promoter usage, and different poly­(A) addition sites, the four human TPM genes (Figure S1) collectively produce more than 40 mRNA variants that can be translated as tissue-specific proteins in skeletal and nonskeletal tissue types.^24,35^

Missense mutations in TPMs are linked to cardiac diseases such as hypertrophic cardiomyopathy (HCM)^25^ and skeletal muscle diseases^25,36–38^ (Figure 1). These mutations may modulate interactions of TPMs with other filament proteins, impacting the contractile apparatus.^39–41^ Altered TPM expression and phosphorylation have also been linked with dilated cardiomyopathy (DCM) and cancer phenotypes.^32,33^ Because TPMs are subject to extensive variation at the genetic, post-transcriptional, and post-translational levels,^42^ the functional diversity and high sequence similarity of TPM proteoforms presents a daunting challenge with current analytical methods.^43^

Therefore, in this study, we sought to determine whether the NGPS could distinguish between diverse TPM proteoforms. We focused on three major types of variants: isobaric peptides from TPM1/2 gene paralogues, peptides derived from tissue-specific TPM2 spliceforms, and phosphotyrosine (pTyr; pY)-modified variants from unmodified peptides. In the process, we leveraged key features of the Platinum sequencing data type: characteristic pulse durations (PDs), which distinguish structurally similar NAAs, and the order of discrete recognition segments (RSs), which pinpoint amino acid location. Together, these measurements produce “kinetic signatures” that provide information about both amino acid identity and sequential order. Given that sequence variations within our model system correspond to regions relevant to TPM pathophysiology (Figure 1), we envision broader applications of NGPS to the targeted detection of proteoform-informative peptides from biological samples.

## Materials and Methods

### In Silico Digestion and Peptide Selection Criteria

Protein sequences of canonical TPM1 and TPM2 spliceforms^44^ (Figure 1) were extracted from GENCODE (v43). In-silico LysC^45^ digestion of TPM sequences was performed using a custom R script leveraging the R package cleaver. The resulting peptide sequences were mapped to their corresponding genomic coordinates using PoGo and visualized as a UCSC browser track to identify peptides that are specific to TPM1 (ENALDRAEQAEADK and VIESRAQK) or TPM2 (ENAIDRAEQAEADK and VIENRAMK), shared between spliceforms of TPM2 (VIENRAMK), and specific to certain spliceforms of TPM2 (SLMASEEEYSTK, TIDDLEETLASAK, and TIDDLEDEVYAQK).

Peptides that are paralogue-specific, shared between TPM2 spliceforms, and TPM2 spliceform-specific were filtered according to the following criteria: the peptide contains a C-terminal lysine (Lys; K), the peptide contains ≥3 amino acids that are cognate to the NAA recognizers, the peptide sequence can be recognized by ≥ 3 different recognizers (Figure S3), and the peptide contains 5–25 amino acids. These criteria are important for successful sequencing of peptides by NGPS (Library Preparation Kit, Lys-C Data Sheet, and Platinum Analysis Software Data Sheet).

### Peptide Synthesis

Eight peptides identified from the in silico peptide screening (Figure 1) were synthesized by JPT Peptide Technologies, Inc. (Berlin, Germany) with C-terminal carboxylic acid and azido-lysine modifications (1 mg, >90% purity, lyophilized). Peptides were reconstituted in 50% acetonitrile to a concentration of 5 mM each and stored at −80 °C.

Synthetic phosphopeptides and unphosphorylated peptides with C-terminal azido-lysine modifications were synthesized by Genscript (Piscataway, NJ, USA) (4 mg, >90% purity, lyophilized). The peptides were reconstituted in dimethylformamide to concentrations of 5 mM each and stored at −80 °C.

### LC–MS/MS Analysis

Peptide separation was performed by using nanoflow high-performance liquid chromatography (HPLC) on a Dionex Ultimate 3000 system (Thermo Fisher Scientific, Bremen, Germany). Peptides were initially loaded onto an Acclaim PepMap 100 trap column (300 μm × 5 mm, 5 μm C18), followed by gradient elution through an Acclaim PepMap 100 analytical column (75 μm × 25 cm, 3 μm C18) for enhanced separation. Mass spectrometry (MS) analysis was conducted using an Orbitrap Eclipse Tribrid mass spectrometer (Thermo Fisher Scientific, Bremen, Germany) equipped with the Orbitrap Eclipse Tune (version 4.0.4091) and Xcalibur software (version 4.5.445.18) for data acquisition and analysis.

### Preparation of Linker-Conjugated Peptide Libraries

Synthetic peptides with C-terminal azido-lysine modifications were conjugated to linker molecules via strain-induced click conjugation.^46^ Peptides were diluted to a final concentration of 50 μM in 100 mM HEPES, pH 8.0 (20% acetonitrile), and incubated overnight with 2 μM linker at 37 °C. For experiments to discriminate between highly similar peptide sequence variants, each conjugated peptide and its paralogous or alternatively spliced counterpart were mixed in equimolar ratio to produce 20 nM conjugated peptide libraries. The resulting conjugated peptide libraries were stored at −20 °C until sequencing.

### Peptide Sequencing on Platinum

Experiments were conducted in accordance with the Library Preparation Kit Lys-C Data Sheet and Platinum Instrument and Sequencing Kit V2 Data Sheet (February 27, 2024).

Library Preparation Kit -Lys-C Data Sheet link: https://www.quantum-si.com/wp-content/uploads/DATA-SHEET_QSI_Library-Prep_V1_DIGITAL.pdf.

Platinum Instrument and Sequencing Kit V2 Data Sheet link: https://www.quantum-si.com/wp-content/uploads/DATA-SHEET_QSI_Sequencing-Kit_V2_Print.pdf/

Briefly, conjugated peptides were immobilized in nanoscale reaction chambers on a semiconductor chip (Figure 2A) for exposure to a mixture of freely diffusing NAA recognizers and aminopeptidases. The mixture consisted of NAA recognizers that target 12 of the 20 canonical AAs (Figure 2B). During on-chip sequencing, NAA recognizers reversibly bind cognate NAAs, producing recognition segments (RSs) that were captured by the semiconductor chip (Figure 2C). During the sequencing process, aminopeptidases cleave the peptide bond and expose the subsequent NAA for recognition. After 10 h of runtime, sequencing data was transferred to the Platinum Analysis Software.

**Figure 2:**
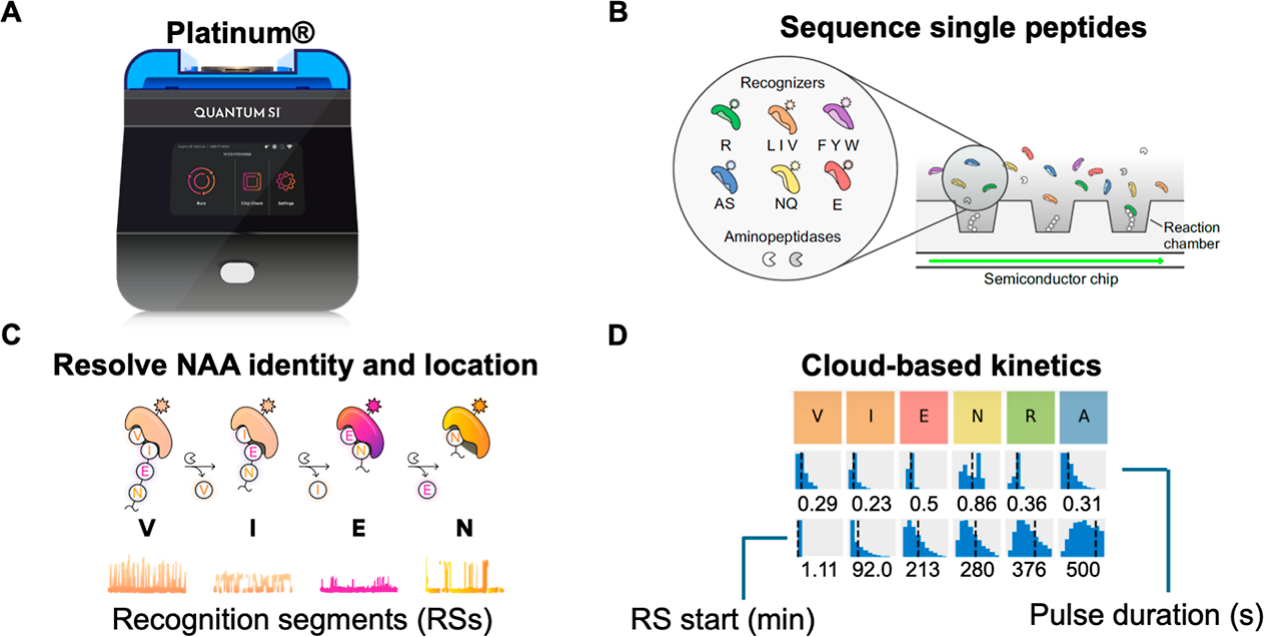
Overview of sequencing on Platinum. (A) The Quantum-Si NGPS platform consists of a semiconductor chip, sample prep and sequencing kits, the compact Platinum instrument (27 lbs), and cloud-based software analysis. (B) The semiconductor chip contains discrete nanoapertures spread across two flow cells that can accommodate single peptide molecules. The sequencing kits employ aminopeptidases and six N-terminal amino acid (NAA) recognizers, which are labeled with different fluorophores. (C) During sequencing, recognizers reversibly bind NAAs, generating characteristic pulsing regions called recognition segments (RSs). As NAA recognizers can target 1–3 NAAs, distinct pulse duration (PD) provides a kinetic readout of NAA identity. (D) Upon completion of a sequencing run, data is automatically transmitted for cloud-based analysis. Reads are then aligned to the reference profile based on the correspondence of observed RSs to the expected reference profile, using recognizer identity. Kinetic signature numerical values (RS start, PD) represent the median of a distribution of data across analyzed apertures that contains 4 RS and 3 unique dyes.

### Cloud-Based Analysis of Sequencing Data: Analysis Versions

Primary Analysis version 2.5.1 and Peptide Alignment version 2.5.2 were used for cloud-based analysis of sequencing data. Details can be found in the Platinum Analysis Software Data Sheet (February 2, 2024): https://www.quantum-si.com/resources/product-data-sheets/platinum-analysis-software-data-sheet/

The fluorescence trace processing and analysis algorithms used by the software have been described in detail in Chinnaraj et al.^47^ and will be briefly summarized here.

The Primary Analysis workflow is the first step in data processing (Figure S3). During the Primary Analysis, distinct pulses of recognizer–peptide interactions are extracted from the raw fluorescence trace collected by the instrument. The traces are segmented to produce recognition segments based on observed transitions in fluorescence intensity and lifetime over the course of the sequencing reaction. The fluorescence intensity and lifetime of each pulse are fitted to a Gaussian Mixture Model to determine the identity of the dye and corresponding recognizer at each recognition segment. In the Peptide Alignment workflow, each segmented read is aligned against the provided reference peptide sequences. For each possible alignment generated between the read and the reference, an alignment score is calculated by using a dynamic programming method that rewards agreement between the observed mean pulse durations at each recognition segment in the read and the expected pulse durations for each amino acid position predicted for the reference sequence. The read is matched to the target peptide sequence, producing the highest alignment score. Finally, a false discovery rate is computed for each aligned reference peptide using a target-decoy approach.

## Results and Discussion

We first sought to identify proteoform-specific peptides in TPM1 and TPM2 to serve as the focus of this study. We performed an in silico digestion of TPM1 and TPM2 with Lys-C, an enzyme that cleaves peptide bonds at the C-terminal side of lysine (Lys; K) residues.^45^ We then selected eight peptides that correspond to distinct regions along the TPM1/2 primary structure (Figure 1). In addition, amino acid mutations in these peptides have been found to be relevant to TPM pathophysiology^25,27–29,32,33^ (Figures 1 and S2).

To discriminate gene paralogues at the peptide level, we tested two sets of paralogous, isobaric peptides by NGPS on Platinum: ENALDRAEQAEADK (TPM1)/ENAIDRAEQAEADK (TPM2) (Figures 1, Peptide Pair A, and 3A) and VIESRAQK (TPM1)/VIENRAMK (TPM2) (Figures 1, Peptide Pair B, and 3C). ENALDRAEQAEADK and ENAIDRAEQAEADK are identical in the amino acid sequence, except for the fourth position from the N-terminus, in which there is an isobaric Leu/Ile substitution (Figures 1, Peptide Pair A, and 3A). In other words, they share the same length and molecular weight, and so, specifically detecting each peptide requires differentiation of signals from Leu and Ile. We conjugated the peptides and mixed the resulting linker-derivatized peptides in an equimolar ratio for loading on both flow cells of the semiconductor chip (Materials and Methods). For comparison of ENALDRAEQAEADK and ENAIDRAEQAEADK, the first three amino acids (Glu1/E1, Asn2/N2, and Ala3/A3) are identical, and each can be measured via distinct recognizers (Figure 2B). The recognizer for Leu, Ile, and Val has the highest affinity for N-terminal Leu, and this preference is expected to result in a markedly longer PD for LDR compared to that for IDR in these two peptides.^21^

**Figure 3:**
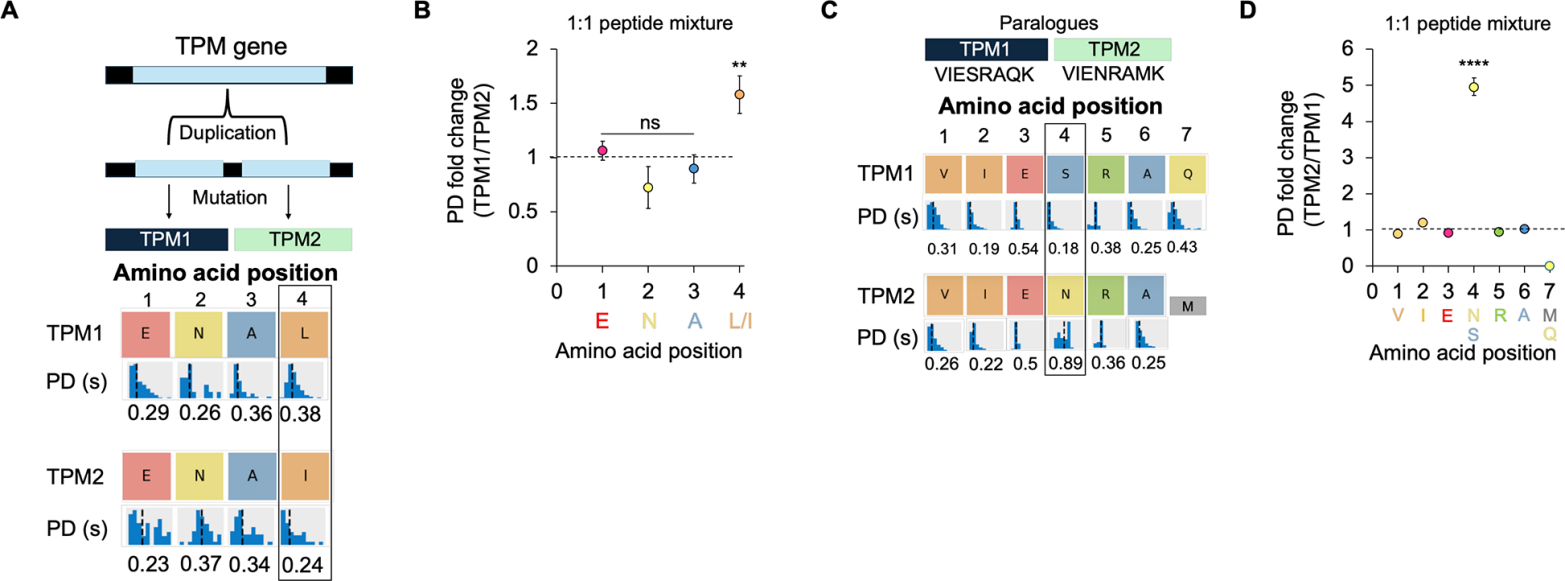
Kinetic signatures discern the identity and amino acid ordering of paralogue-derived peptides. (A) Gene duplication events produce TPM1/TPM2 paralogues, wherein a single isobaric Leu to Ile substitution differentiates ENALDRAEQAEADK from ENAIDRAEQAEADK (top). An example kinetic signature snapshot from cloud-based analysis is shown below the amino acid positions (bottom). Colors of the boxes indicate the recognizer identity (see Figure 1B). Kinetic signatures such as PD represent the statistical distribution of kinetic data for all pulses associated with a specific residue. (B) Plot of the fold change in the average pulse duration (PD) between NAA measurements of ENAL versus ENAI, for the first 4 residues. Error bars indicate standard deviation from three experiments conducted on independent flow cells. Asterisks indicate *p*-values relative to a PD fold change of 1. (C) Gene duplication events produce TPM1/TPM2 paralogues that can be differentiated at position 4 of TPM1 peptide VIESRAQK and TPM2 peptide VIENRAMK. (D) Plot of the fold change PDs for VIENSRAQ versus VIENRAM. A ∼5-fold higher PD was detected for the Asn in position 4 of VIENRAMK, relative to Ser in VIESRAQK, which was statistically significant, when compared to the PD fold change to all other positions. Data (*mean ± S.D.) are representative of three independent experiments, conducted on independent flow cells (ns *p* > 0.05, *p < 0.05, **p < 0.01, ***p < 0.001, ***p < 0.0001; two-tailed *t*-test). PD = Pulse Duration.

Indeed, we observed a higher average PD for Leu (0.35 s) compared with that for Ile (0.23 s) (Figure 3B). These results indicate a single recognizer distinguishes isobaric residues that differ at position 4 from the N-terminus, thus confidently discriminating these paralogue-specific peptides.

Interestingly, we observed similar trends of highly specific peptide recognition with the paralogous peptides VIENRAMK and VIESRAQK, in which all residues are the same except for the Asn to Ser substitution in position 4 and the Met to Gln substitution in position 7 (Figures 1, Peptide Pair B, and 3C). While all matched residues between the two peptides exhibit similar PD profiles, the Asn to Ser substitution at position 4 was distinguished via the dye call for each recognizer (Figure 3C). In addition, the recognizers for Asn and Ser elicited different PDs for their cognate residues, providing an additional metric for distinguishing each residue at this position. Indeed, an ∼5-fold longer PD was detected for Asn relative to Ser (Figure 3D).

In addition to paralogues, the TPM gene family also produces alternatively spliced isoforms that are tissue specific and functionally distinct. In TPM2, exon 6 or 7 is included, but not both, with the same mutually exclusive splicing of exons found for exons 10 and 11. We found that SLMASEEEYSTK maps to a specific splice junction that overlaps exon 6 and thus could inform the presence of the spliceform TPM2-210, which has been found to be expressed in nonskeletal muscle^48–51^ (Figures 1, Peptide Pair C, and 4A). From the sequencing of SLMASEEEYSTK, PDs were generated for all amino acids within the peptide except for the Met at position 3 (Figure 4B).

**Figure 4:**
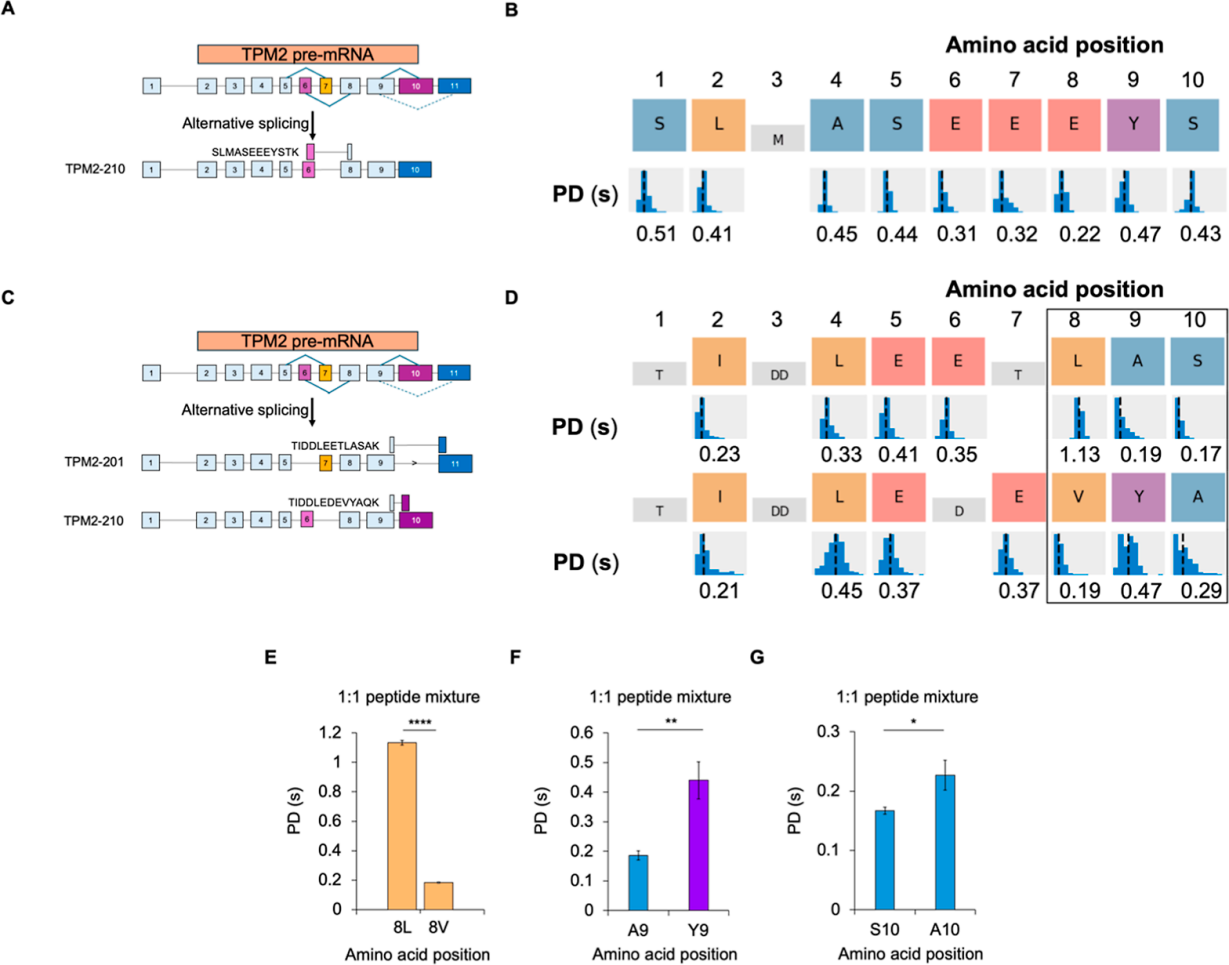
Kinetic signatures discern the identity and temporal order of TPM2 peptides derived from **alternative splicing**. (A) Alternative splicing of TPM2 mRNA generates a tissue-specific spliceform (containing Exon 6) to which SLMASEEEYSTK specifically maps. (B) Sequence alignments for SLMASEEEYSTK. An example kinetic signature snapshot from cloud-based analysis is shown below the amino acid positions. Colors of the boxes indicate the recognizer identity (see Figure 1B). Kinetic signatures such as PD represent the statistical distribution of kinetic data for all pulses associated with a specific residue. (C) Alternative splicing of TPM2 mRNA differentiates exon-specific peptides TIDDLEETLASAK (Exon 10) and TIDDLEDEVYAQK (Exon 11). (D) Sequence alignments for peptides TIDDLEETLASAK and TIDDLEDEVYAQK. (E) LIV recognizer elicits differential PD profiles for similar aliphatic residues. A ∼9-fold difference in PD was observed for Leu/Val. (F) Distinct recognizers for Ala and Tyr discern position 9. (G) The Ala/Ser recognizer enables discernment via differential PDs at position 10. Data (*mean ± S.D.) are representative of three independent experiments, conducted on independent flow cells (ns *p* > 0.05, *p < 0.05, **p < 0.01, ***p < 0.001, ***p < 0.0001; two-tailed *t*-test). PD = Pulse Duration.

We also found a peptide pair that discriminates exon 10 from exon 11, with exon-specific peptides of the same length: TIDDLEETLASAK (exon 11) and TIDDLEDEVYAQK (exon 10) (Figures 1, Peptide Pair D, and 4C). The combination of residue position, recognizer identity, and PD led to a highly specific discrimination of the peptides (Figure 4D). In particular, residues in positions 8–10 were clearly distinguished by recognizer and PD patterns. For position 8—in an additional demonstration of the ability of the LIV recognizer to discern aliphatic amino acids (Figure 3B)—we observed a ∼9-fold higher PD for the Leu relative to valine (Val; V) (Figure 4E). In position 9, Tyr and Ala residues were clearly distinguished with distinct recognizers (Figure 4F). In further support of the ability of a single recognizer to elicit differential PD profiles, we observed a higher PD for Ala relative to Ser in position 10 (Figure 4G). Taken together, these results demonstrate specific sequencing of splice junction-specific peptides with NGPS and demonstrate that NGPS differentiates peptides based on both structurally similar and distinct amino acid side chains.

Finally, we tested the ability of NGPS to distinguish peptidoforms^52^ arising from PTMs—a major source of proteoform variation. One of the benefits of single-molecule sequencing is discrete binding events between each recognizer and its cognate amino acid, which is a highly sensitive modality of detection. We found that the previously sequenced peptide TIDDLEDEVYAQK (Figure 4), which is specific to exon 10 of TPM2, contains an annotated phosphorylation at the Tyr in position 10.^53^ The addition of a phosphate group significantly changes the charge and topology of Tyr, weakening binding by the recognizer for bulky aromatic residues (Figure 1B).

We separately conjugated TIDDLEDEVYAQK and TIDDLEDEVpYAQK (Figure 5A) and then loaded each peptidoform for on-chip sequencing in independent flow cells. As expected, we observed a significant decrease in percent of total nanoapertures containing Tyr (Y) RSs for the phosphorylated form relative to the unmodified form, while the percent of nanoapertures containing Leu/Ile/Val (LIV) and Glu (E) RSs was similar between the two groups (Figure 5B). These results indicate that Platinum sequencing is sensitive to the presence of PTMs; thus, kinetic signatures can be used to infer the presence of PTMs at single amino acid resolution.

**Figure 5:**
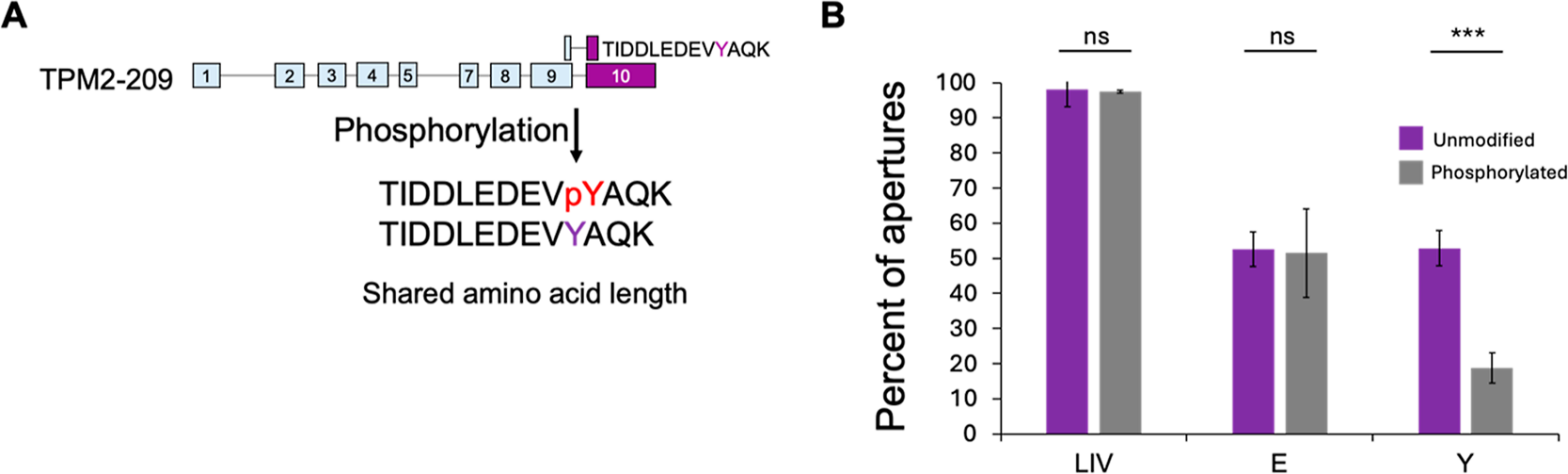
YFW recognizer generates Y-specific recognition events, enabling differentiation from pY-modified peptidoforms. (A) Schema showing the exon-specific peptide TIDDLEDEVYAQK, which contains a known phosphosite at the Tyr residue. (B) Bar plot showing the difference in percent of apertures between the recognizers (LIV, E, and YFW) in the unmodified versus phosphorylated version (TIDDLEDEVpYAQK). Data (mean ± S.D.) are representative of three independent experiments, conducted on independent flow cells (ns *p* > 0.05, ***p < 0.001; Welch two-sample *t*-test).

## Conclusions

Analyzing the proteome is essential for a comprehensive understanding of biological processes. However, several fundamental challenges hinder the ability to fully characterize the proteomic diversity. These challenges stem from the complexity of the proteome, which could contain over 1 million distinct proteoforms.^1,14^

Existing methods for detecting proteoform-informative peptides, such as mass spectrometry, can struggle with distinguishing closely related variants, particularly those that differ by single amino acid substitutions or PTMs. These methods can have other limitations, including high costs and detection thresholds as well as the requirement for specific expertise to operate and interpret MS experiments. In contrast, by directly sequencing individual peptides in an accessible benchtop format, NGPS has the potential to overcome some of the limitations of traditional methods, serving as an orthogonal tool for proteomic discovery.

In this study, we sought to determine the ability of the benchtop Platinum instrument to distinguish proteoform-informative peptides derived from the human proteome. To select a target protein family suited for the assessment of the capability of NGPS to distinguish peptide variants, we screened protein families for peptide variants that passed specific criteria required for NGPS (see Materials and Methods for criteria). In addition, to ensure the variant differences could be theoretically captured by NGPS, we also considered whether the amino acid substitutions, splice changes, or PTM sites could be recognized by the current set of recognizers. The TPM protein family proved to be an ideal model for our assessment, as it contains numerous proteoforms with peptide variants suitable for detection by NGPS and is implicated in a range of diseases (Figure 1). Using this single-molecule protein sequencing technology, we identify key variations at the paralogue, transcript, and PTM levels. While prior work^21^ laid the foundation for the detection of amino acid variation using NGPS, this study represents the first application of NGPS for sequencing proteoform-informative peptides.

We demonstrate the ability of NGPS to differentiate between paralogous TPM1 and TPM2 peptides that differ by a single amino acid substitution as well as to sequence tissue-specific TPM2 splice variants. As TPMs are subject to post-transcriptional regulation (e.g., long noncoding RNA and antisense transcripts),^11,54^ there is a need to validate TPM expression at the peptide/protein level. In future studies with biological samples, a pan-TPM antibody that recognizes a conserved site on each TPM isoform could be used for upfront enrichment, followed by NGPS to identify peptides that uniquely map to each TPM variant. An intriguing extension of our study would be to apply NGPS to differentiate sequence variants resulting from repeat expansion mutations, such as polyglutamine expansions in the huntingtin protein—key to the pathology of Huntington’s disease.^55^ With single amino acid resolution, NGPS may distinguish these variants by counting distinct Gln recognition events within individual peptide reads. Continued development of analysis software to better resolve successive cleavages and recognition of identical amino acids will be critical for advancing this promising application. More generally, given its orthogonal chemistry, Platinum can provide additional evidence to support peptide identifications via DDA from MS (Figure S4A,B).

Our findings also illustrate the sensitivity of NGPS to phosphotyrosine modifications at the single amino acid level. Given this capability, future studies could use NGPS to perform target identification for phospho-specific antibodies.^56^ Detecting phosphorylation is particularly challenging due to the transient nature of these modifications and the complex interplay of signaling networks, making high-resolution methods essential for capturing these dynamic processes.^57^ More broadly, our findings further demonstrate the sensitivity of NGPS to PTMs, building on prior evidence of its ability to distinguish between Met and oxidized Met^21^ and detect Arg modifications^58^ through PTM-induced changes in binding. Together, these results suggest that NGPS can differentiate a wide range of PTMs based on their effects on recognizer binding kinetics. Future studies that systematically characterize how diverse PTMs influence binding could enable a broader application of NGPS to PTM identification, including the development of software trained to detect characteristic kinetic signatures associated with specific modifications.

There are several limitations of this study. First, we chose to focus on proteoform-informative synthetic peptides as a model system for this foundational proof-of-concept evaluation. Thus, future studies will be required to extend these findings to biological samples. Second, because we used either 1:1 mixtures of peptides (Materials and Methods) or individual peptides loaded in independent flow cells (Y and pY), future studies with complex mixtures will be required to simulate conditions typically obtained from proteomic studies. Higher complexity could present analytical challenges, such as reduced sequencing depth, which could reduce the confidence of detection of specific kinetic differences between peptide variants. Beyond this study, several innovations will help expand the biological applications of NGPS, including the development of additional recognizers to expand the sequencing coverage and scaling of the nanowell capacity of the chip to increase read counts. Recently, Quantum-Si released a combined Asp/Glu recognizer; as tropomyosin proteoforms contain an abundance of acidic residues (Figures 1 and S5), additional peptides could likely be resolved by differentiating acidic sites with the latest sequencing kit. We are continuing to investigate the implementation of new developments in NGPS to distinguish peptide variants within protein isoform mixtures.

Broadly, the application of NGPS for proteoform differentiation can be generalized to other proteoform families. While TPMs have been greater than 500 million years in the making,^39^ new TPM discoveries are on the horizon, with not only mammals but also six TPM genes that have been found in zebrafish, a popular animal model for gene expression studies.^59^ In addition, TPMs are the primary allergens in shellfish, causing food allergies that affect 2% of the US adult population.^60,61^ Hence, detecting TPM peptides that elicit food allergies could be of great interest to the food science community.^43^

More generally, NGPS on Platinum could integrate with existing proteogenomic approaches to identify immunopeptides derived from noncoding regions,^62,63^ enabling the detection of functional biomolecules often overlooked by conventional methods. Further, combining NGPS with genomic and transcriptomic data could provide a more comprehensive view of proteoform diversity, including low-abundance variants and post-translational modifications. This multiomics strategy will lead to an improved understanding of the proteome in health and disease.

## Supplementary Material











## Data Availability

Mass spectrometry data from Skyline were uploaded to Panorama and are publicly available (ProteomeXchange ID: PXD057918; Panorama public link: https://panoramaweb.org/Ye3utk.url). Kinetic signatures for each peptide obtained from sequencing with Platinum are shown in Tables 1–4. Platinum sequencing data used in this work are available on Zenodo (https://zenodo.org/records/15298902).

## Notes

Exonic structure of the human tropomyosin genes, MS2 spectra and fragmentation map of 7 synthetic TPM peptides, schematic of Platinum library preparation, chip loading, and peptide alignment, recombinant TPM2 subjected to endoproteinase Lys-C and LC–MS/MS (Orbitrap Eclipse), and TPM1/2 amino acid frequency of NAA recognizers compatible with Platinum (PDF)
Kinetic signatures obtained from sequencing the equimolar mixture of peptides ENALDRAEQAEADK and ENAIDRAEQAEADK, kinetic signatures obtained from sequencing the equimolar mixture of peptides VIENRAMK and VIESRAQK, kinetic signatures obtained from sequencing the peptide SLMASEEEYSTK, and kinetic signatures obtained from sequencing the equimolar mixture of peptides TIDDLEETLASAK and TIDDLEDEVYAQK (XLSX)
Overview of the Library preparation process using the Library Preparation Kit, Lys-C; specifications of Library Preparation Kit, Lys-C: specifications for protein samples; proteins with various sizes tested with the Library Preparation Kit, Lys-C; sequencing results of proteins across a wide range of molecular weights prepared by the Library Preparation Kit, Lys-C; performance of the Library Preparation Kit, Lys-C at input concentrations of 1–5 μM for IL6, CDNF, IL4, and PDL1; overview of the Library preparation process; and sequencing results of immunoprecipitated IL6 compared with recombinant IL6, both prepared with the Library Preparation Kit, Lys-C; (PDF)
Overview of the sequencing process on Platinum, increase in amino acids detected with sequencing kit V2 across various proteins, cohorts for intra-chip reproducibility studies, and assessment of performance between two sides of the sequencing chip across different sample cohorts based on numbers of alignments (PDF)
Overview of the protein sequencing setup using Platinum analysis software, primary analysis characterizing apertures based on loading, recognizer activity, and recognizer read lengths, and peptide alignment and false discovery rate profiles (PDF)
