# Supplementary material for: Protein Sequencing with Single Amino Acid Resolution Discerns Peptides That Discriminate Tropomyosin Proteoforms
Source: J Proteome Res. 2025 Jun 10;24(8):3798–807. doi: 10.1021/acs.jproteome.4c00978 (PMC12322949; doi:10.1021/acs.jproteome.4c00978)
Supplement: Supplementary file 1 [file pr4c00978_si_001.pdf]

Protein Sequencing with Single Amino Acid Resolution Discerns Peptides That Discriminate Tropomyosin Proteoforms

Natchanon Sittipongpittaya[1#], Kenneth A. Skinner[4#*], Erin D. Jeffery[1], Emily F. Watts[1], Gloria M. Sheynkman[1,2,3,*]

[1]Department of Molecular Physiology and Biological Physics, University of Virginia, Charlottesville, Virginia 22903, United States

[2]Department of Biochemistry and Molecular Genetics, University of Virginia, Charlottesville, Virginia 22903, United States

[3]UVA Comprehensive Cancer Center, University of Virginia, Charlottesville, Virginia 22903, United States

[4]Quantum-Si Incorporated, 29 Business Park Drive, Branford, Connecticut 06405, United States

#Equal contribution

*Co-corresponding author email addresses: kskinner@quantum-si.com; gs9yr@virginia.edu

Table of Contents

Supplementary Figure 1 2
Supplementary Figure 2 3
Supplementary Figure 3 4
Supplementary Figure 4 5
Supplementary Figure 5 6
Supplementary Tables 1-4 .... XLSX

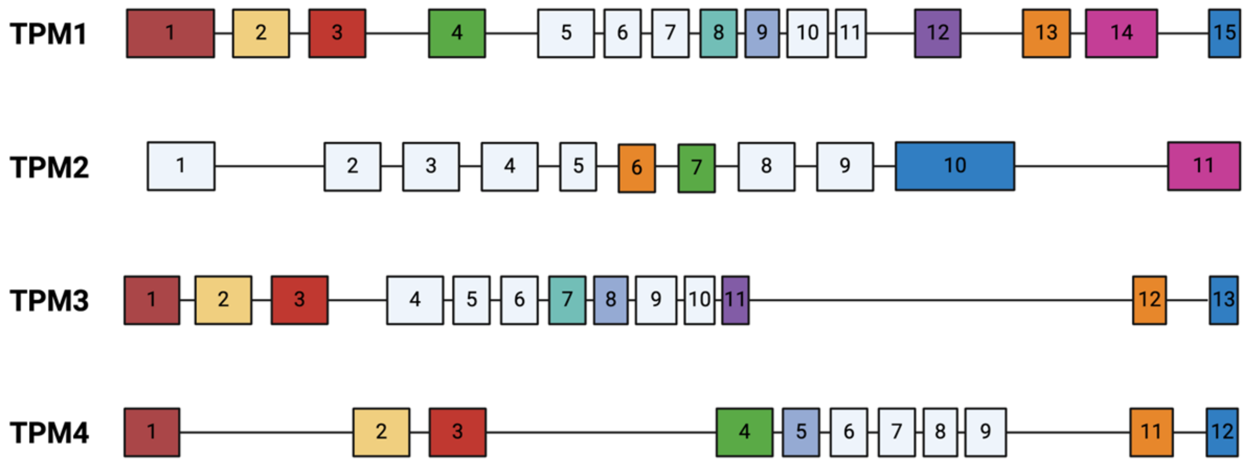

Supplementary Figure 1. Exonic structure of the human tropomyosin genes. Most vertebrates have four tropomyosin genes, each encoding multiple isoforms due to alternate exon expression. Each tropomyosin gene is composed of 11 to 15 exons, with different splicing patterns correlated with different functions. The *white* boxes are constitutively expressed in all spliceforms of the gene, while *colored* boxes are alternatively spliced to produce distinct spliceforms. The most variable regions of tropomyosin are the ends, encoded by alternate start and end exons that form the intermolecular overlap junction.

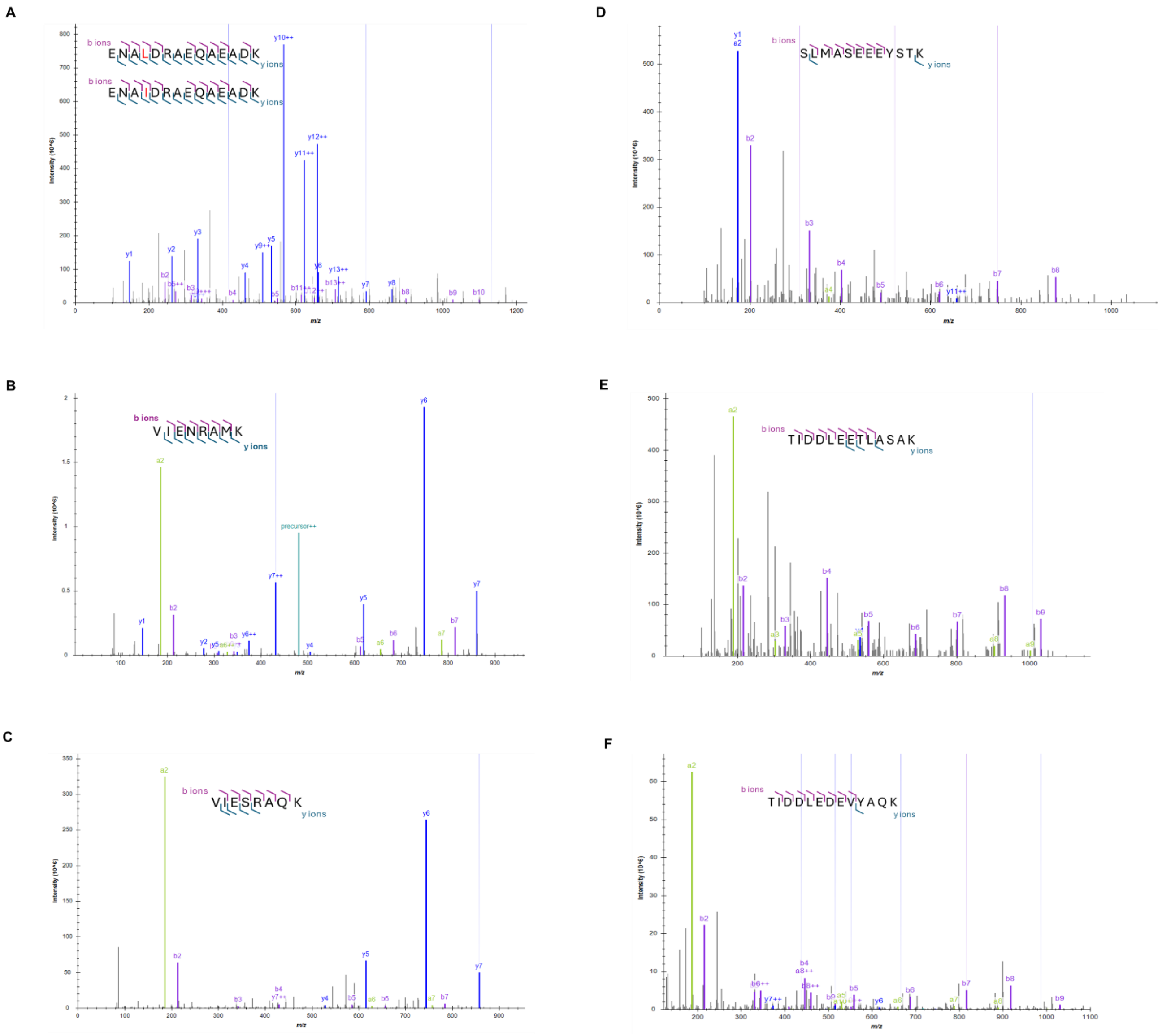

Supplementary Figure 2. MS2 spectra and fragmentation map of 7 synthetic TPM peptides. A-F shows the ion intensities and fragmentation of each peptide: (**A**) ENA[L/I]DRAEQAEDK (spectra are indistinguishable from each other), (**B**) VIENRAMK, (**C**) VIESRAQK, (**D**) SLMASEEEYSTK, (**E**) TIDDLEETLASAK, (**F**) TIDDLEDEVYAQK

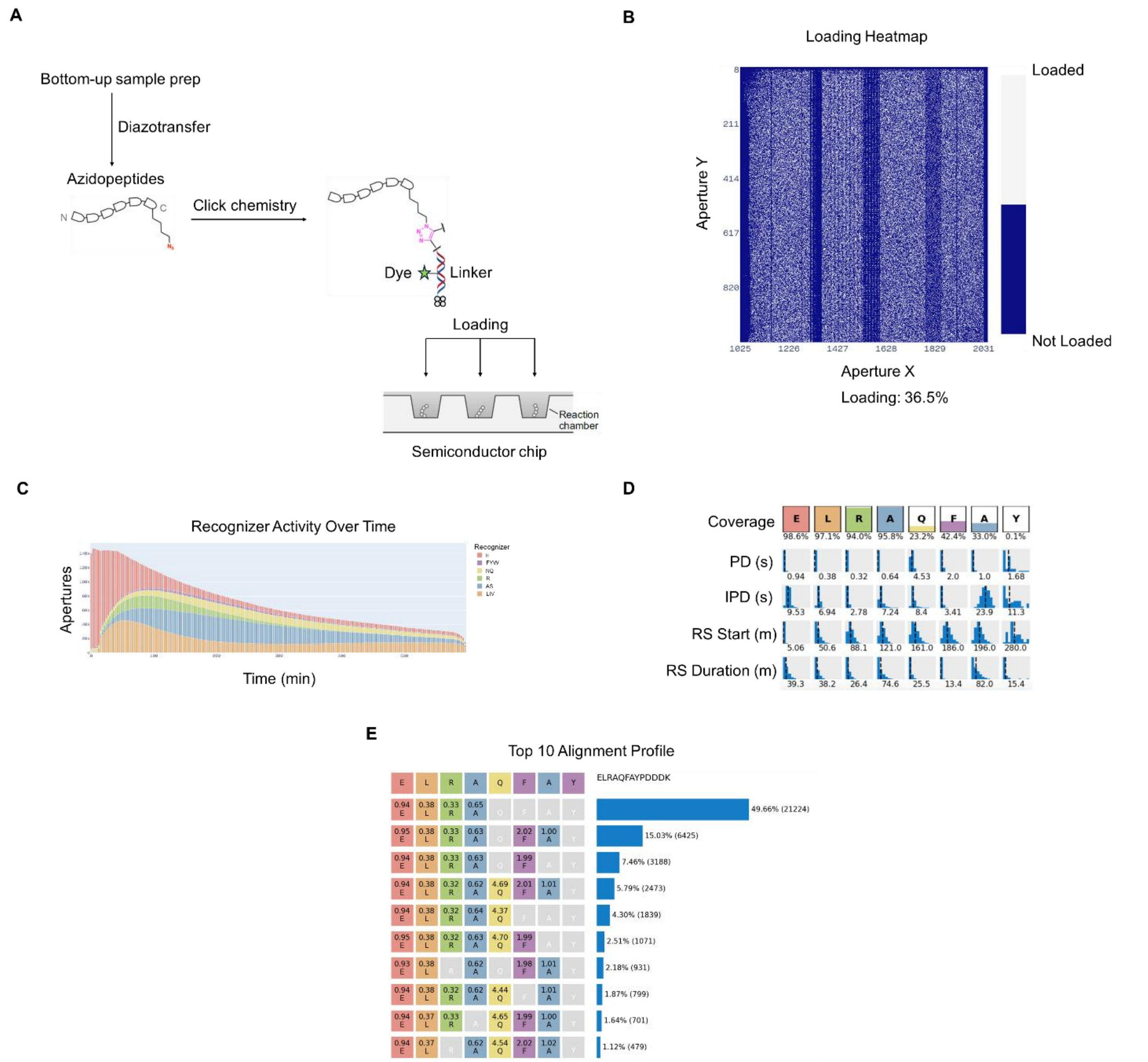

Supplementary Figure 3. Schematic of Platinum library preparation, chip loading, and peptide alignment. (**A**) Peptides are subjected to diazotransfer to install an N_3 group at the side chain of the C-terminal lysine. The derivatized peptides are conjugated to fluorescently labeled linkers via click chemistry, then loaded onto flow cells on a semiconductor chip and immobilized in nanoscale reaction chambers (or apertures). (**B**) A heatmap of aperture occupancy is generated after on-chip peptide loading. White pixels represent loaded apertures, while dark blue pixels are unoccupied apertures. (**C**) Recognizer activity over the course of a sequencing experiment. Number of apertures registering pulsing activity for each recognizer type are recorded and plotted in real-time. (**D**) Example coverage map of a synthetic peptide ELRAQFAYPDDK. Colored boxes show the frequency of detection for each residue. The distribution and median of each of the kinetic parameters collected by Platinum are displayed as blue histograms underneath each residue. (**E**) Reads are grouped into different alignment profiles based on the RSs they exhibit. The number of reads in each alignment profile for this peptide are displayed as blue bars to the left of the profiles.

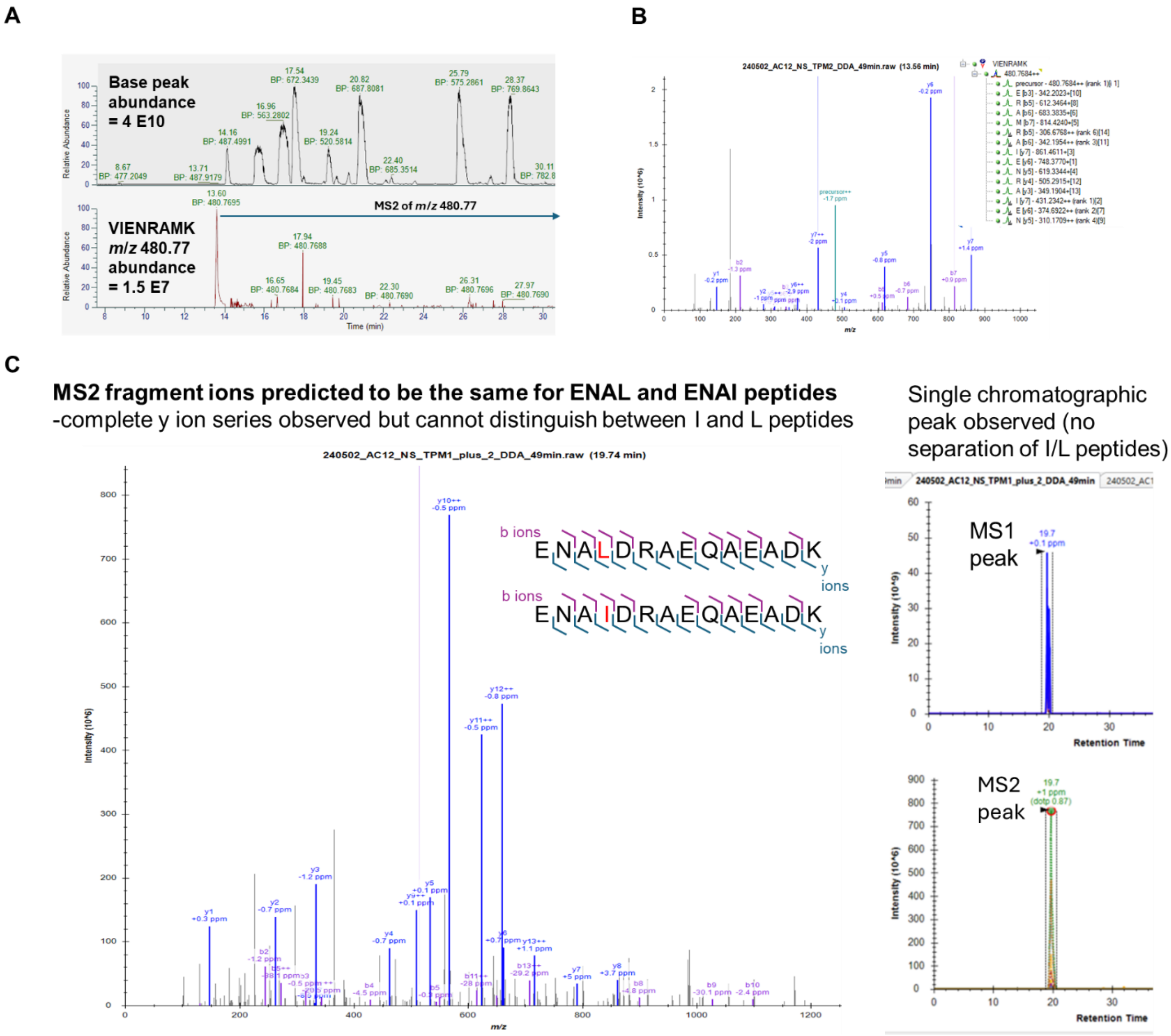

Supplementary Figure 4. Recombinant TPM2 subjected to endoproteinase Lys-C and LC-MS/MS (Orbitrap Eclipse). (**A**) VIENRAMK peptide ion abundance is 1000-fold less than base peak abundance, leading to a single MS2 scan acquired during DDA method. (**B**) A spectrum of VIENRAMK with b/y ion series. (**C**) Single chromatographic peak observed with isobaric peptides ENALDRAEQAEADK (TPM1) and ENAIDRAEQAEADK (TPM2) complicates LC-MS/MS detection of paralog-specific peptides.

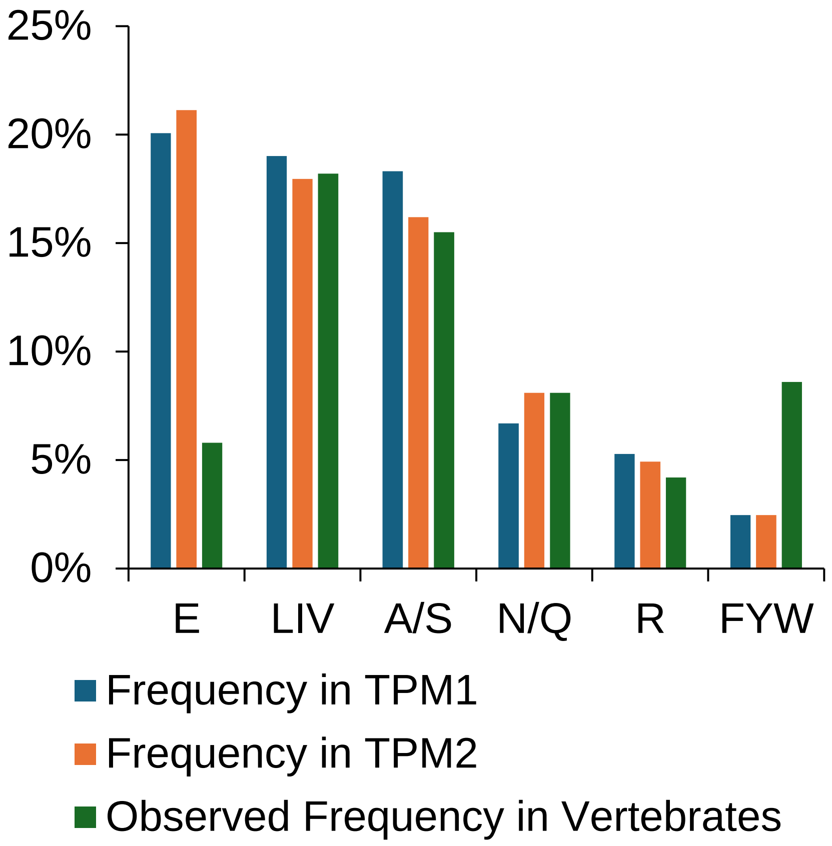

Supplementary Figure 5. TPM1/2 amino acid frequency of NAA recognizers compatible with Platinum.
