# Supplementary material for: Protein Sequencing with Single Amino Acid Resolution Discerns Peptides That Discriminate Tropomyosin Proteoforms
Source: J Proteome Res. 2025 Jun 10;24(8):3798–807. doi: 10.1021/acs.jproteome.4c00978 (PMC12322949; doi:10.1021/acs.jproteome.4c00978)
Supplement: Supplementary file 3 [file pr4c00978_si_003.pdf]

Library Preparation Kit - Lys-C Data Sheet

Easily Prepare Proteins for Next-Generation Protein Sequencing™ on Platinum® in a Simple User-Friendly Workflow

Feburary 27, 2024

INTRODUCTION

Platinum®, the world's first Next-Generation Protein Sequencer™, delivers single-molecule and single-amino acid resolution in a user-friendly benchtop platform. Platinum enables protein identification and variant detection without complex workflows and advanced expertise, making new discoveries possible for every lab.

The Platinum instrument, kits, and software contain everything you need to prepare, sequence, and analyze proteins. Library preparation is the initial step in Quantum-Si's Next-Generation Protein Sequencing™ workflow. The Library Preparation Kit - Lys-C (Catalog # 910-00012-00) contains the necessary components to digest proteins into peptides and functionalize and conjugate peptides for immobilization on a semiconductor chip. In the library preparation process (Figure 1), intact proteins first undergo reduction and alkylation to break disulfide bonds and cap free thiol groups. The proteins are then digested at the carboxyl side of lysine residues using the endoprotease Lys-C. The resulting peptides are functionalized at the C-terminal lysine residues with azide groups for conjugation to a macromolecular K-linker via click chemistry. The peptide libraries are then ready for chip immobilization and sequencing with Sequencing Kits on the Platinum instrument. Dye-labeled N-terminal amino acid (NAA) recognizers bind on-off NAA residues and aminopeptidases cleave each NAA exposing the next NAA for recognition until the entire peptide is sequenced. Fluorescent intensity, lifetime, and binding kinetics data from each NAA binding event and the order of recognition make up kinetic signatures for each peptide which can be automatically transferred to the Platinum Analysis Software for peptide alignment and protein identification.[1]

For Research Use Only. Not for use in diagnostic procedures.

FIGURE 1. OVERVIEW OF THE LIBRARY PREPARATION PROCESS USING THE LIBRARY PREPARATION KIT - LYS-C.

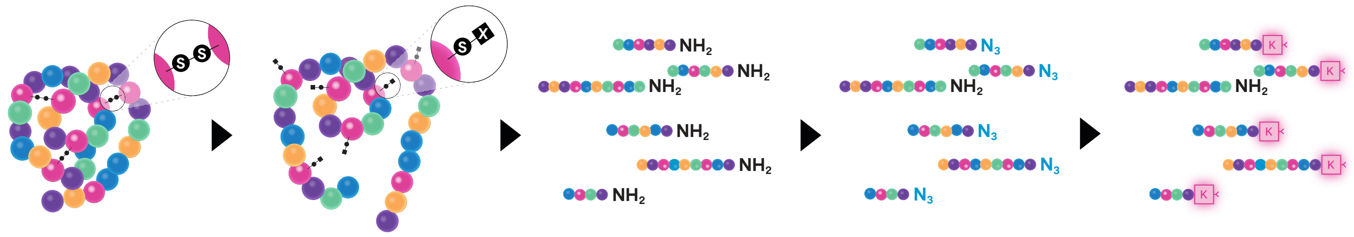

The performance of Library Preparation Kit - Lys-C was evaluated on various sample types, on samples at various input concentration, and on proteins with a wide range of molecular weights (Table 1). Peptide and protein sequencing data of various sample types demonstrates the utility of the Library Preparation Kit - Lys-C for proteins with various molecular weights, concentrations, and number of peptides, as well as the flexibility in modifying the library preparation protocol for immunoprecipitated proteins and synthetic peptides.

TABLE 1. SPECIFICATIONS OF THE LIBRARY PREPARATION KIT - LYS-C SPECIFICATIONS FOR PROTEIN SAMPLES.

Sample Types	Purified recombinant proteins, immunoprecipitated proteins, protein mixtures containing up to 10 proteins
Libraries per Kit	8
Recommended Input Amount	100 µL of 5 µM solution
Molecular Weights Tested	11–67 kDa
Number of LysC-Digested Peptides (≥ 3 residues) per Protein Tested	11–53 peptides
Average Length of Digested Peptide	13 residues
Library Preparation Time	Hands on time: < 3 hours<br>Total time: 2 days

PROTEIN IDENTIFICATION OF PROTEINS WITH A RANGE OF MOLECULAR WEIGHTS DEMONSTRATES ROBUSTNESS

The average molecular weight of a protein in the human proteome is around 50 kDa.[2] To demonstrate the performance of the Library Preparation Kit - Lys-C on proteins with a range of molecular weights spanning the average size, 9 different proteins ranging in molecular weight from 11 to 67 kDa were each prepared twice. When digested with Lys-C, these proteins produced 11–53 peptides each

For Research Use Only. Not for use in diagnostic procedures.

peptide containing 3 or more residues and averaging in length of about 13 residues per peptide (Table 2). Subsequently, each resulting library underwent sequencing twice on Platinum with Sequencing Kit V2 (Catalog # 910-00011-00), totaling 4 runs for each protein. Protein identification was completed by aligning kinetic signatures to peptide sequences with predictable kinetic signatures using the Peptide Alignment analysis workflow. The number of alignments per protein is determined by the composition of each protein, specifically the concentration of peptides with at least 4 amino acids recognized by at least 3 distinct recognizers. Confidence in peptide alignment and protein identification can be evaluated by the false discovery rate (FDR) reported for each peptide within a protein.

TABLE 2. 10 PROTEINS WITH VARIOUS SIZES TESTED WITH LIBRARY PREPARATION KIT - LYS-C

Protein	Uniprot ID	Sequence of Tested Construct	Molecular Weight (kDa)	Number of LysC-Digested Peptides (≥ 3 residues))
H4	P62805	Full-Length	11.4	11
IL4	P05112	Met + H25-End	15.1	13
FGF2	P09038	Ala + P143-End	16.5	14
CDNF	Q49AH0	Q25-End + 10His	19.9	17
IL6	P05231	P29-End	20.9	13
PDL1	Q9NZQ7	Start-T239	27.6	15
VIME	P08670	S2-End	53.5	21
AKT1	P31749	6His + FLAG + Full-Length	57.5	33
HSA	P02768	D25-End	66.5	53

All 9 proteins were successfully identified on Platinum with the average number of alignments per protein ranging from 447 (FGF2) to 19,420 (CDNF) (Figure 2A) and the average number of peptides with FDR ≤ 10% ranging from 2.25 peptides (AKT1) to 7 peptides (HSA) (Figure 2B). Differences in the observed number of alignments and high-quality peptides can be attributed to the protein's composition. For example, proteins with a low number of lysine residues will not generate as many peptides for sequencing, and proteins with less visible residues (residues that are not detectable by the current sequencing chemistry) will result in less recognition events and less alignments. These data demonstrate that a variety of proteins differing in molecular weight and amino acid composition can be confidently identified over a broad range of overall number of alignments when high-quality low FDR peptides are aligned to proteins of interest.

For Research Use Only. Not for use in diagnostic procedures.

FIGURE 2. SEQUENCING RESULTS OF PROTEINS ACROSS A WIDE RANGE OF MOLECULAR WEIGHTS PREPARED BY THE LIBRARY PREPARATION KIT - LYS-C.

A) Molecular Weight of Each Protein vs. the Number of Alignments in log scale. B) Number of Peptides of each protein vs the Number of High-Quality Peptides Detected (< 10% FDR)

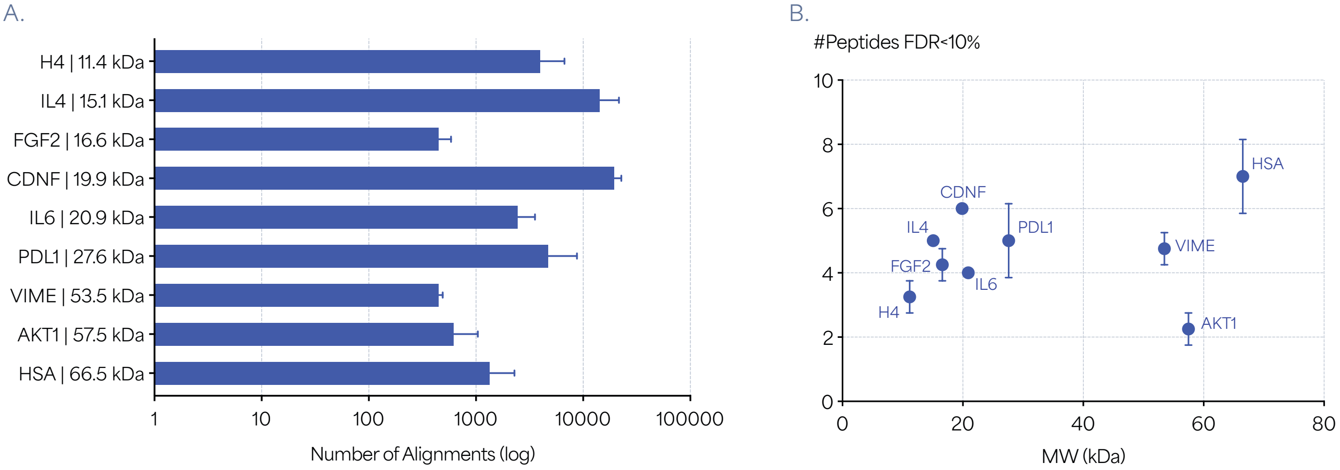

ADJUSTING PROTEIN INPUT AMOUNT SHOWCASES CORRELATION BETWEEN CONCENTRATION AND NUMBER OF ALIGNMENTS

Proteins exist at various concentrations in nature. Understanding how protein sequencing performance is impacted by different protein input amounts will provide guidance on assay optimization and results interpretation. The current library preparation protocol recommends an input concentration of 5 µM. Other concentrations (2.5 µM and 1 µM) were tested to evaluate the performance of the Library Preparation Kit - Lys-C at lower input concentrations. Specifically, interleukin-4 (IL4), interleukin-6 (IL6), cerebral dopamine neurotrophic factor (CDNF), and programmed death-ligand 1 (PDL1) were prepared using 5 µM, 2.5 µM, and 1 µM input concentrations and sequenced on Platinum.

Sequencing data demonstrated a correlation between input concentrations and the number of alignments (Figure 3). Libraries prepared at 1 µM input produced the lowest numbers of alignments for all four proteins. Libraries prepared with 1 µM and 2.5 µM protein input concentration respectively produced on average 21–43% and 36–70% of the number of alignments of libraries prepared with 5 µM protein input concentration (Figure 3A). Nevertheless, the libraries prepared with 1 µM and 2.5 µM protein input generated sufficient alignments to the same peptides with FDR < 10% as the libraries prepared with 5 µM protein input (IL6: 4 peptides; PDL1: 4–6 peptides; IL4: 5 peptides; CDNF: 6 peptides) (Figures 2B and 3B). These results demonstrated that the four proteins tested were still aligned to their respective reference at an input concentration 5x lower than the protocol recommends, and there is a correlation between protein input amount and number of alignments.

FIGURE 3. PERFORMANCE OF THE LIBRARY PREPARATION KIT – LYS-C AT INPUT CONCENTRATIONS OF 1–5 µM FOR IL6, CDNF, IL4, AND PDL1, ASSESSED BY NUMBER OF ALIGNMENTS FROM PLATINUM SEQUENCING.

A) Number of alignments normalized by the average values obtained with libraries prepared at 5 µM input. B) Total number of alignments.

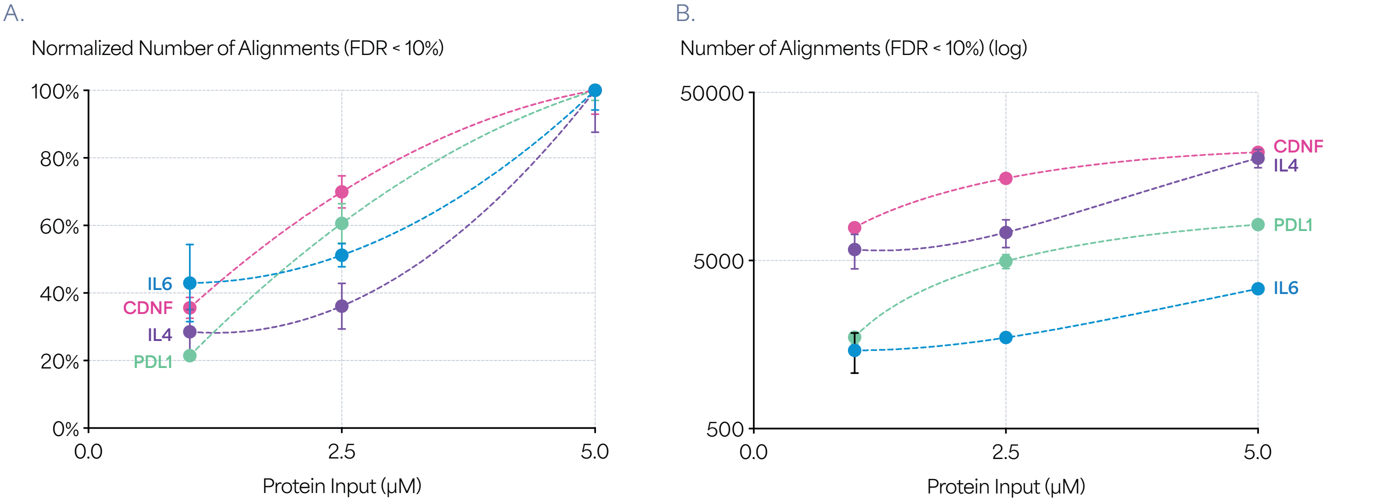

PROTEIN IDENTIFICATION AND INFERENCE OF IMMUNOPRECIPITATED FROM BIOLOGICAL SAMPLES

Studying proteins from biological samples is key to understanding human health and disease. Demonstrating the compatibility of the Library Preparation Kit – Lys-C with common immunoprecipitation workflows enables new applications on Platinum for studying key proteins in cells and other biological samples. To sequence immunoprecipitated proteins, the library preparation protocol needs to be modified so that reduction, alkylation, and digestion occur on beads (Figure 4).[3] Dynabeads™ M-270 Streptavidin (ThermoFisher, Cat. No. 65305) are recommended for immunoprecipitation, but other resins such as Streptavidin Agarose (ThermoFisher, Cat. No. 20353) or Protein A Agarose (ThermoFisher, Cat. No. 20333) have also been tested.

FIGURE 4. OVERVIEW OF THE LIBRARY PREPARATION PROCESS ON PROTEINS IMMUNOPRECIPITATED FROM BIOLOGICAL SAMPLES.

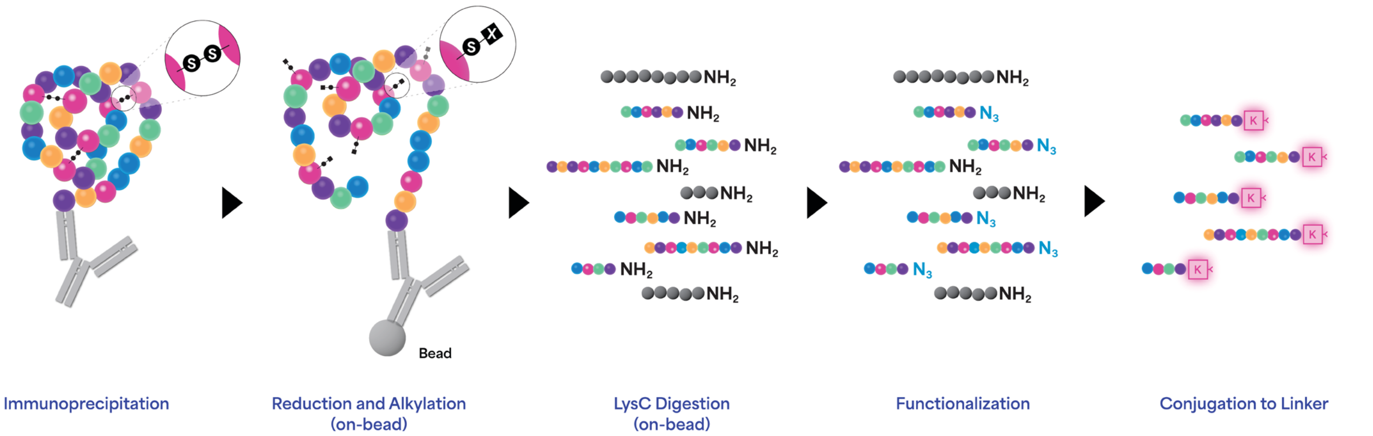

To demonstrate the ability to prepare immunoprecipitated proteins with Library Preparation Kit - Lys-C, IL6 was introduced into human serum, subjected to immunoprecipitation via an on-bead digestion procedure, and subsequently sequenced 8 times on Platinum. The sequencing result was compared with that of recombinant IL6. As shown in Figure 5, the IL6-IP library generated an average of 3,675 alignments, very similar to an average of 3,399 alignments of recombinant IL6. Both recombinant IL6 and IL6-IP library aligned to 3–4 IL6 peptides with FDR ≤ 10% using the Peptide Alignment Workflow. This result demonstrates that library preparation of immunoprecipitated IL6 generates similar sequencing results and protein identification as recombinant IL6 protein.

FIGURE 5. SEQUENCING RESULTS OF IMMUNOPRECIPITATED IL6 COMPARED WITH RECOMBINANT IL6, BOTH PREPARED WITH LIBRARY PREPARATION KIT - LYS-C.

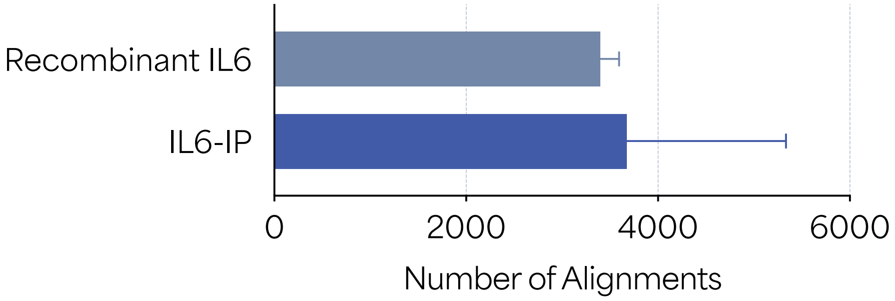

Additionally, the immunoprecipitated IL6 protein was analyzed using the Protein Inference Workflow.[4] This workflow aligns proteins to a large panel of thousands of proteins and infers the likelihood that certain proteins are present in the sample without being known. To demonstrate the use of this workflow on the immunoprecipitated IL6 protein, sequencing data was analyzed using a panel of 7,921 proteins with a molecular weight of 10–70 kDa, and with at least three in silico LysC-digested peptides with three unique, visible residues. The inferred proteins are presented in a table, with their ranking determined by their respective Inference Score, calculated from the number of alignments and FDR of all digested peptides (Table 3). The results indicated that IL6 was inferred as the protein most likely present in the sample, with an Inference Score of 12.83, in contrast to 8.61 for the second inferred protein. It is important to note that the Inference Score is a natural log representation of the FDR associated with the inferred protein. Therefore, the score difference of 4.22 between IL6 and the second inferred protein corresponds to approximately a 68-fold difference in the ratio of FDR between the second inferred protein and IL6. This data suggests that protein inference can be used in applications studying unknown immunoprecipitated proteins, such as testing antibody specificity or co-immunoprecipitated proteins.

$$\textit{Inference Score(protein)} = -\ln(\textit{FDR(protein)})$$

$$\Delta \textit{Score} = \textit{Inference Score}(\textit{protein}_1) - \textit{Inference Score}(\textit{protein}_2) = \ln \frac{\textit{FDR}(\textit{protein}_2)}{\textit{FDR}(\textit{protein}_1)}$$

TABLE 3. SAMPLE RESULTS FROM PROTEIN INFERENCE ANALYSIS WORKFLOW ON IMMUNOPRECIPITATED IL6.

Inference Score and ΔScore metrics are natural log calculations of the FDR of proteins.

Inference Rank	Protein	Uniprot ID	Inference Score (Protein)	Number of Peptides with FDR ≤ 10%	Number of Alignments
1	IL6	P05231	12.83	3	980
2	LRC28	Q86X40	8.62	2	361
3	OR8J2	Q8NGG4	5.67	1	289
4	PCP	P42785	5.31	1	203
5	OR5H6	Q8NGV6	5.21	1	31

FLEXIBILITY IN SAMPLE INPUT TYPES ENABLES USE OF PEPTIDES AS STARTING MATERIAL FOR LIBRARY PREPARATION

Peptides play crucial regulatory roles in cellular processes, serve as biomarkers or potential drug candidates, or serve as "barcodes" to simplify protein selection and screening.[5] Demonstrating the compatibility of the Library Preparation Kit - Lys-C with peptides as starting materials instead of intact proteins opens new ways for exploring the significance of peptide sequences in health, disease, and therapeutic research using Platinum. To sequence individual synthetic peptides or peptide mixtures, the library preparation protocol is modified so that the C-terminal azidolysine residue of peptides are directly conjugated with the K-linker without the need for reduction, alkylation, digestion, and functionalization of the protein.

To evaluate the robustness of the Library Preparation Kit - Lys-C on peptides, we prepared two libraries of the same mixture of 10 azido-terminated peptides (peptides synthesized with C-terminal azidolysines) with distinguishable amino acid sequences (Figure 6A). Each library was sequenced on Platinum at least eight times, and the average numbers of alignments for each peptide in the mixture were compared between the two libraries. As shown in Figure 6B, the two libraries generated very similar numbers of alignments for all 10 peptides. This result demonstrates sequencing reproducibility of two separately prepared libraries of a 10-peptide mixture.

For Research Use Only. Not for use in diagnostic procedures.

FIGURE 6. SEQUENCING PERFORMANCE, ASSESSED BY NUMBER OF ALIGNMENTS, OF TWO DIFFERENT LIBRARIES OF THE SAME MIXTURE OF 10 AZIDO-TERMINATED PEPTIDES.

A.

QP155 - ELRAQFAYPDDDK

QP335 - FQRIALNFAK

QP586 - FAQLQARFAADDDK

QP634 - ARLAFAYPDDDK

QP706 - VRFLEQQNK

QP746 - LRYAFAYPDDDK

QP796 - EFLNRFYK

QP798 - ENRLCYYLGATK

QP1027 - RLAIQFAYPDDDK

QP1088 - DQFRLAGGK

B.

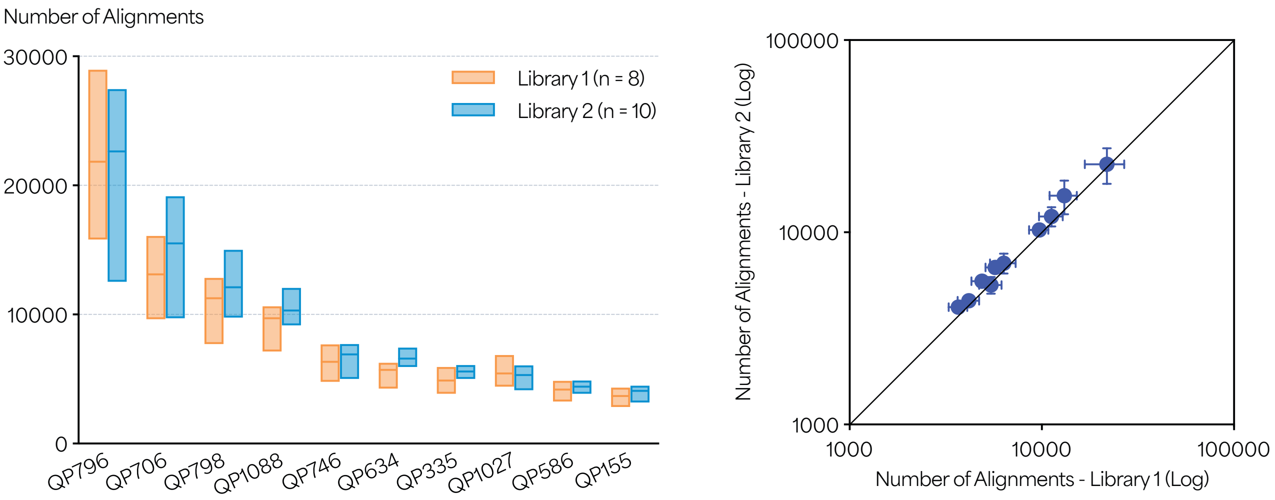

SUMMARY

The Library Preparation Kit – Lys-C enables researchers to easily prepare proteins and peptides with different molecular weights and peptide compositions for next-generation protein sequencing on Platinum. Different concentrations of protein input correlate to differences in peptide alignments, and the library preparation protocol is amenable to other starting material such as immunoprecipitated proteins and peptide mixtures. Researchers can integrate this simple workflow into other laboratory workflows for easily preparing protein samples and generating groundbreaking research through protein identification and variant detection with single-molecule resolution on Platinum. For more information, visit www.quantum-si.com/products/.

For Research Use Only. Not for use in diagnostic procedures.

ORDERING INFORMATION

Product	Catalog Number
Library Preparation Kit - Lys-C	910-00012-00

RELATED PRODUCTS

Product	Catalog Number
Sequencing Kit v2.0	910-00011-00
Platinum® Instrument	910-10904-00
Premium Service Contract	700-00005-00
Basic Service Contract	700-00006-00
Advanced Next-Generation Protein Sequencing™ Training	700-00004-00
Sequencing Control Peptide, SDQP155	910-00013-00

REFERENCES

1. Reed, B. D. et al. Real-time dynamic single-molecule protein sequencing on an integrated semi-conductor device. Science 378, 186–192 (2022).
2. Hendil, K. B., Hartmann-Petersen, R., Tanaka, K. 26 S proteasomes function as stable entities. J Mol Biol 315, 627–636 (2002).
3. Application Note: Immunoprecipitation of IL-6 from Human Serum for Next-Generation Protein Sequencing on Platinum®.
4. Data Sheet: Platinum® Software Analysis Data Sheet.
5. Application Note: Peptide Barcodes for Next-Generation Protein Sequencing™.

QUANTUM SI™

29 Business Park Drive, Branford, CT 06405

www.quantum-si.com | 866.688.7374
