# Supplementary material for: Protein Sequencing with Single Amino Acid Resolution Discerns Peptides That Discriminate Tropomyosin Proteoforms
Source: J Proteome Res. 2025 Jun 10;24(8):3798–807. doi: 10.1021/acs.jproteome.4c00978 (PMC12322949; doi:10.1021/acs.jproteome.4c00978)
Supplement: Supplementary file 4 [file pr4c00978_si_004.pdf]

Platinum® Instrument and Sequencing Kit V2 Data Sheet

Sequence Proteins with Single-Molecule and Single-Amino Acid Resolution on Platinum® using New Sequencing Kit V2 Enhancements

Feburary 27, 2024

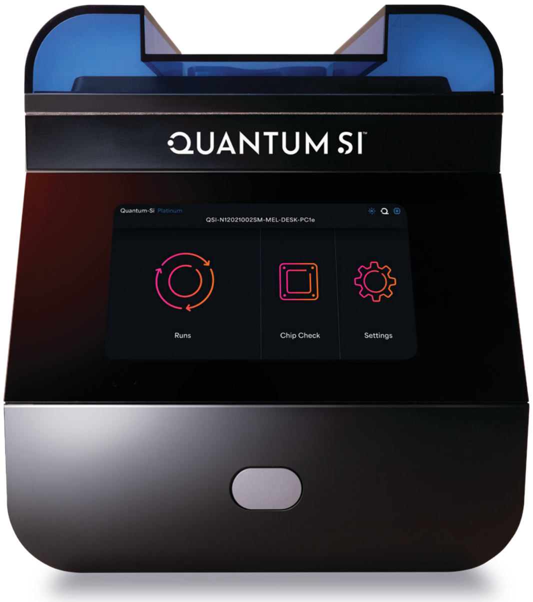

INTRODUCTION

Platinum®, the world's first Next-Generation Protein Sequencer™, delivers single-molecule and single-amino acid resolution in a user-friendly benchtop platform. Platinum enables protein identification and variant detection without complex workflows and advanced expertise, making proteomics accessible for every lab.

For Research Use Only. Not for use in diagnostic procedures.

Quantum-Si's Platinum instrument, kits, and software contain everything you need to prepare, sequence, and analyze proteins. Proteins are digested into peptide libraries using the Library Preparation Kit – Lys-C. Peptides are immobilized onto the sequencing chip, followed by the addition of sequencing reagents containing a mixture of dye-labeled N-terminal amino acid (NAA) recognizers and aminopeptidases. The sequencing process commences as the recognizers interact with each NAA and generate a fluorescent signal from which the binding kinetics characteristic of each amino acid are extracted (Figure 1A). Specifically, as the recognizers repeatedly associate and dissociate with the NAAs, a distinct series of pulses, termed a recognition segment (RS) (Figure 1B), is generated for each recognized NAA with characteristic fluorescence and kinetic properties. Aminopeptidases in solution sequentially remove individual NAAs, exposing subsequent residues for recognition until the entire peptide is sequenced. The temporal order of NAA recognition and associated kinetic properties are analyzed with the Primary Analysis workflow, available with Platinum Analysis Software. To maintain a low false discovery rate (FDR), the Peptide Alignment and Protein Inference workflows, also available in the Platinum Analysis Software, consider only apertures with at least 4 RSs and 3 distinct recognizers for alignment to peptides from reference proteins.[1]

Platinum and Sequencing Kit V2 enable new protein identification and characterization applications with enhanced sequencing performance and new analysis methods for studying unknown proteins.

FIGURE 1. OVERVIEW OF THE SEQUENCING PROCESS ON PLATINUM.

A) Illustration of the sequencing process, depicting how a single peptide is immobilized in a chip well, NAA recognizers are excited, and NAAs are recognized and cleaved until the entire peptide is sequenced. B) Sample aperture-level sequencing data from a single peptide, showing ten distinct recognition segments, representing ten amino acids. Since this peptide produces at least 4 RSs from at least 3 recognizers, it can be used for alignment.

A.

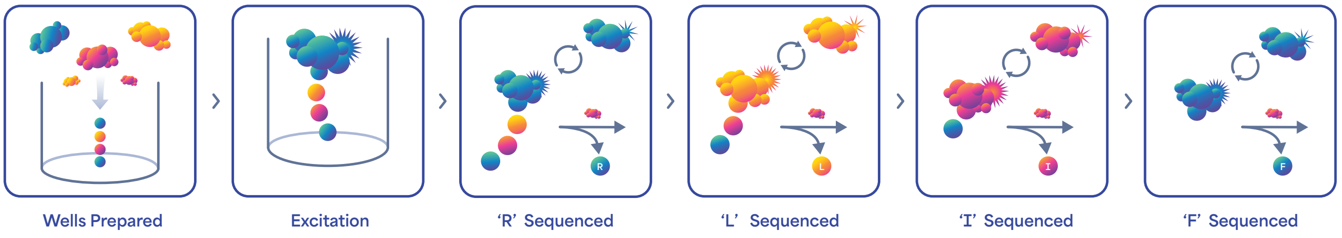

B.

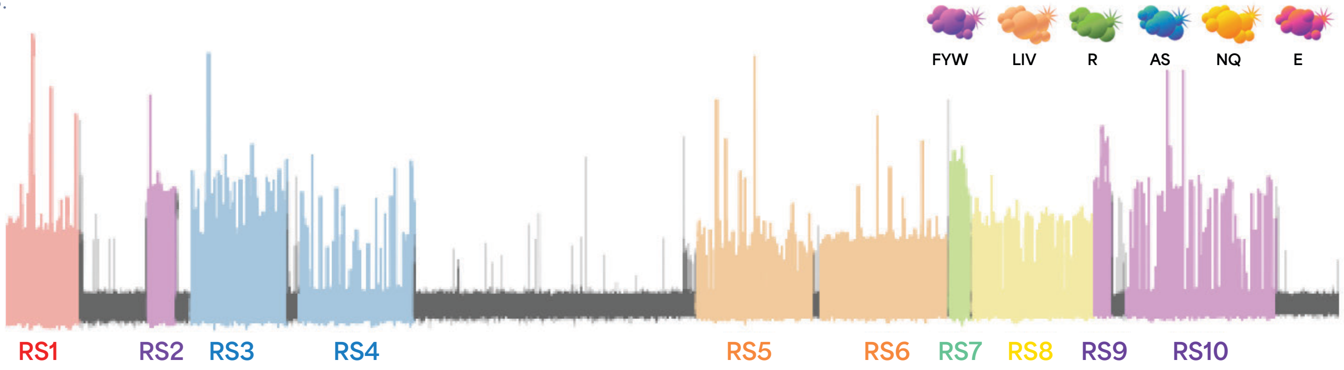

For Research Use Only. Not for use in diagnostic procedures.

SEQUENCING KIT V2 ENHANCES AMINO ACID RECOGNITION FOR PROTEIN IDENTIFICATION AND CHARACTERIZATION

The Sequencing Kit V2 (Catalog # 910-00011-00) is the second generation of sequencing reagents and chips designed for use with Platinum. Key improvements to the recognizers, aminopeptidases, and sequencing chips enhance protein sequencing performance on Platinum. The Sequencing Kit V2 includes an additional recognizer capable of recognizing glutamic acid (E) in addition to the five recognizers included in Sequencing Kit V1 (LIV, FYW, R, AS, and NQ). This additional recognizer along with improvements to the chip and reagent chemistry increase the performance of the Sequencing Kit V2 by delivering an approximately 3-fold increase in amino acids detected per chip compared to V1. Ongoing enhancements in Platinum's performance, as demonstrated with the Sequencing Kit V2, continue to unlock new applications for the use of single-molecule protein sequencing to identify and characterize proteins.

To demonstrate an increase in amino acid recognition on Platinum using the Sequencing Kit V2 compared to V1, the total number of RSs was estimated for four sequencing runs on four different proteins. Specifically, interleukin-4 (IL4), interleukin-6 (IL6), cerebral dopamine neurotrophic factor (CDNF), and programmed death-ligand 1 (PDL1) were selected, as these proteins together exhibit an amino acid diversity similar to that found in the human proteome. As shown in Figure 2, results from protein sequencing runs of these 4 proteins using the Sequencing Kit V2 detected an average of 530,000 amino acids per chip, demonstrating an approximate 3-fold increase in amino acids detected compared to V1.

FIGURE 2: INCREASE IN AMINO ACIDS DETECTED WITH SEQUENCING KIT V2 ACROSS VARIOUS PROTEINS.

The number of amino acids detected per chip was estimated based on the number of RSs detected.

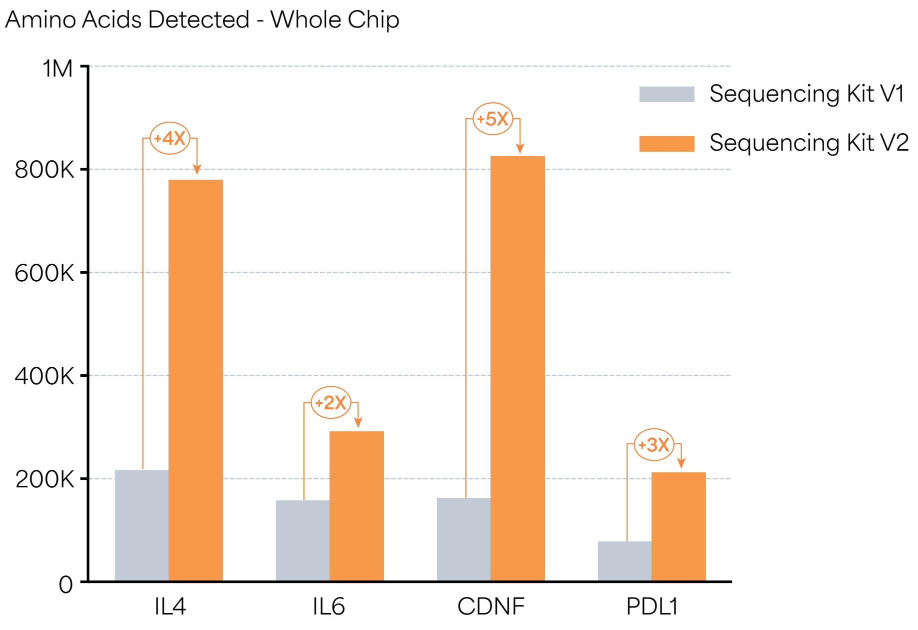

For Research Use Only. Not for use in diagnostic procedures.

INTRA-CHIP REPRODUCIBILITY ENABLES FLEXIBLE EXPERIMENTAL DESIGN ACROSS 2 SIDES OF THE CHIP

The sequencing chip features two separate sides (called flow cells), providing users with the flexibility to load either two libraries simultaneously for increased throughput (split-chip run) or one library on both sides for expanded coverage (whole-chip run) (Figure 3A). To evaluate the sequencing performance between the left and right side of the chip, the number of peptide alignments was assessed for both chip sides for three sample cohorts across 47 chips (Table 1).

TABLE 1. COHORTS FOR INTRA-CHIP REPRODUCIBILITY STUDIES

Cohort	# of Libraries	Library Composition	Total # of Chips
Single Peptide	1	Single Peptide	20
10-Peptide Mix	1	10 different peptides mixed at equimolar ratio	9
Recombinant Proteins	18	9 different recombinant proteins prepared individually, resulting in 2 libraries for each protein	18

To assess performance between chip sides across different cohorts, a paired t-test for two distributions was performed to calculate the p-values of the number of alignments between the left and right sides of the chip. As shown in Table 2, the p-values for the number of alignments for all cohorts were higher than 0.05, suggesting that no significant bias between chip sides was observed. The variability in the number of alignments across the cohorts is determined by the composition of the sample, specifically the concentration of peptides with 4 RSs from 3 distinct recognizers.

TABLE 2. ASSESSMENT OF PERFORMANCE BETWEEN TWO SIDES OF THE SEQUENCING CHIP ACROSS DIFFERENT SAMPLE COHORTS BASED ON NUMBERS OF ALIGNMENTS.

The p-values were assessed using a paired t-test for the two distributions, and a p-value of < 0.05 suggests a bias.

Cohort	# of Libraries	Number of Alignments		
		Median L	Median R	p-Value
Single Peptide	20	141,524	142,662	0.78
10-Peptide Mix	9	91,286	93,084	0.53
Recombinant Proteins	18	1,527	1,598	0.53

For Research Use Only. Not for use in diagnostic procedures.

To demonstrate a correlation between left and right side on individual chips, left side vs right side alignments were plotted for each chip from each cohort (Figure 3B). For the peptide cohorts, each data point represents one chip. For the recombinant protein cohort, each data point represents the average of the two chips for the indicated proteins. The data demonstrates a strong correlation between left and right-side alignments, which enables flexibility to design experiments that compare samples between the two sides of the chip.

FIGURE 3. REPRODUCIBILITY BETWEEN TWO SIDES OF THE SEQUENCING CHIP ACROSS THREE DIFFERENT SAMPLE COHORTS (SINGLE PEPTIDE, 10-PEPTIDE MIX, AND RECOMBINANT PROTEIN).

A) Schematic representation of the sequencing chip showing two sides (flow cells). B) Number of alignments between the two sides of the chip across three different sample cohorts.

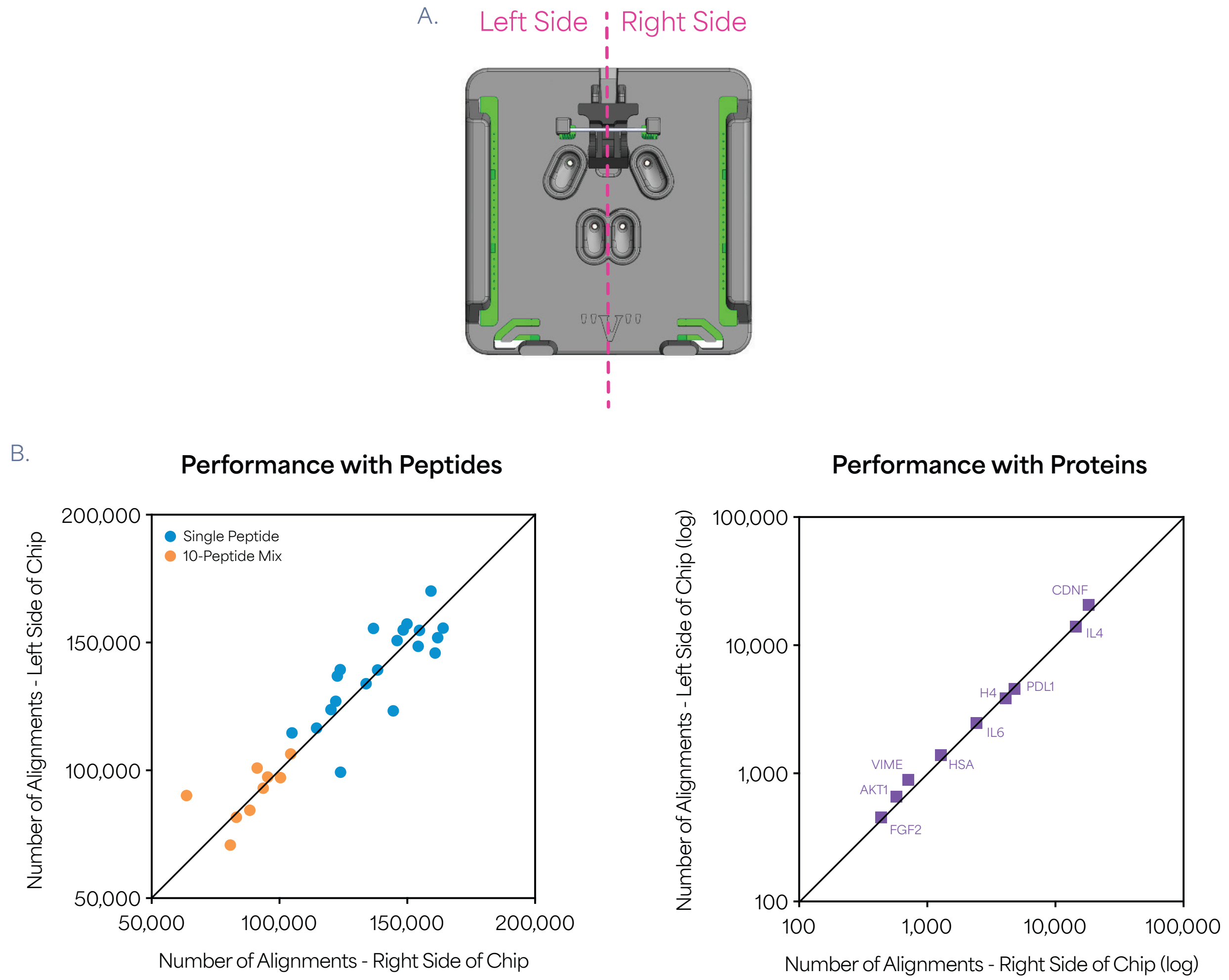

Sequencing Kit Data Sheet.indd 5 2/28/24 1:37 PM

PROTEIN INFERENCE WORKFLOW EMPOWERS RESEARCH APPLICATIONS FOR UNKNOWN PROTEINS

The significant enhancement in sequencing performance with Sequencing Kit V2 on Platinum has facilitated the introduction of a new analysis workflow designed for inferring proteins without prior sequence knowledge. Named the Protein Inference Workflow, this approach is applicable for researchers exploring protein samples that may have unknown proteins,[2] such as protein mixtures, immunoprecipitated proteins,[3] or resolving protein bands from SDS-PAGE.[4] In this workflow, protein sequencing data is aligned to any reference set of proteins. Inferred proteins are then ranked as the most probable protein in the sample by an Inference Score which is calculated from the number of alignments and FDR of all digested peptides. Unlike proteomic methods which require large panels of affinity-based reagents to identify proteins, inference performance on Platinum will be influenced by the selection of the reference set and is not limited to a panel of proteins.

Quantum-Si has developed a pre-defined reference set of 7,921 human proteins which can be used for inferring proteins from unknown samples. The 7,921 proteins have a mass in the range of 10–70 kDa and contain at least three in silico LysC-digested peptides with three unique, visible residues. To demonstrate the use of protein inference, sequencing data from the 9 different proteins from the "Recombinant Protein" cohort specified in Table 1 were sequenced and analyzed using Protein Inference with the reference panel containing 7,921 proteins. An example of the data output from Protein Inference on the IL4 sample is illustrated in Table 3, where the rank order of the inferred proteins is determined by their respective Inference Score. The results indicate that IL4 was correctly inferred as the protein most likely present in the sample, with an Inference Score of 26.11, in contrast to 12.21 for the second inferred protein. It is important to note that the Inference Score is a natural log calculation of the FDR associated with the inferred protein. Therefore, the score difference of 13.90 between IL4 and the second inferred protein (ΔScore = 13.90) corresponds to approximately a 1-million-fold higher FDR of the second protein relative to the first.

$$\textit{Inference Score(protein)} = -\ln(\textit{FDR(protein)})$$

$$\Delta \textit{Score} = \textit{Inference Score}(\textit{protein}_1) - \textit{Inference Score}(\textit{protein}_2) = \ln \frac{\textit{FDR}(\textit{protein}_2)}{\textit{FDR}(\textit{protein}_1)}$$

TABLE 3. SAMPLE RESULTS FROM PROTEIN INFERENCE ANALYSIS WORKFLOW ON IL4.

Inference Rank	Protein	Uniprot ID	Inference Score (Protein)	Number of Peptides with FDR ≤ 10%	Number of Alignments
1	IL4	P05112	26.11	5	6267
2	PP1R7	Q15435	12.21	2	281
3	FEM1C	Q96JP0	9.51	2	176
4	NUP62	P37198	9.22	2	552
5	CACO2	Q13137	8.77	2	599

For Research Use Only. Not for use in diagnostic procedures.

Sequencing Kit Data Sheet.indd 6 2/28/24 1:37 PM

All 9 proteins, sequenced 4 times each, were correctly inferred as the top-ranking protein in every single run with ΔScore values of > 2.30, indicating that the second ranked protein has an FDR that is at least 10 times higher than the top-ranking protein (Figure 4). This result demonstrates that the Protein Inference workflow can effectively infer single proteins and can be applied to research applications where inferring unknown proteins is important, such as immunoprecipitated proteins,[3] or unknown protein bands from SDS-PAGE.[4]

FIGURE 4. DIFFERENCE IN INFERENCE SCORE BETWEEN THE FIRST AND SECOND INFERRED PROTEINS (ΔSCORE) FOR 9 RECOMBINANT PROTEINS (4 RUNS EACH) AS ANALYZED WITH PROTEIN INFERENCE WORKFLOW.

ΔScore ≥ 2.30 indicates a 10-fold difference in confidence level between the first and second inferred proteins.

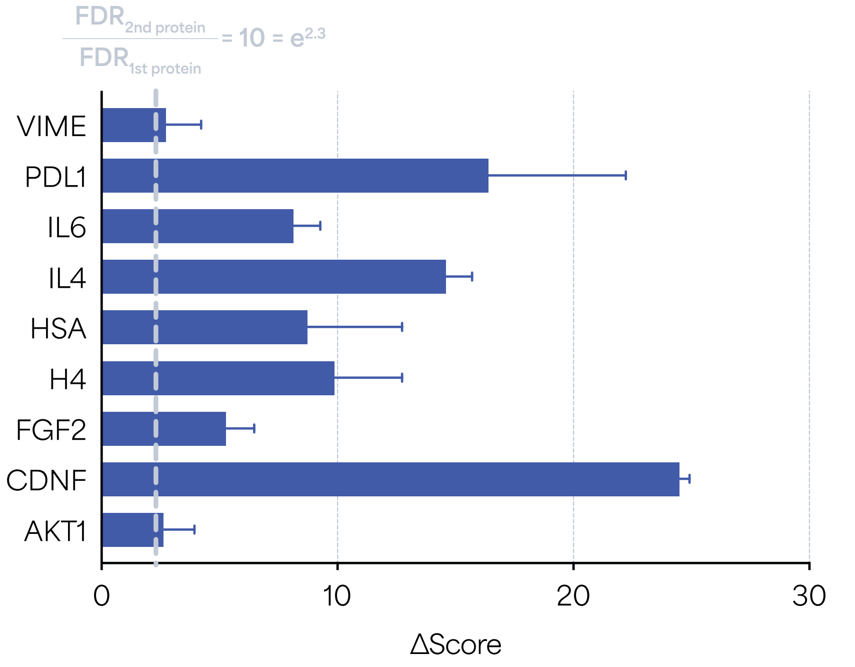

Validating antibody specificity is one application where protein inference can be used to determine if the antibody effectively enriches for the specific protein of interest without specifically indicating which protein to look for. To demonstrate that proteins immunoprecipitated from biological samples can be correctly identified with the Protein Inference workflow, IL6 was introduced into human serum, subjected to immunoprecipitation via an on-bead digestion procedure,[3] and subsequently sequenced on Platinum. Following this protocol, two libraries of immunoprecipitated IL6 (IL6-IP) were prepared, each sequenced 8 times on Platinum. In all 16 runs, IL6 was correctly identified as the top-ranking protein. Furthermore, as shown in Figure 5, the average ΔScore of the IL6-IP runs is ~3.45, which corresponds to a ~32-fold difference in confidence level between IL6 and the second inferred protein. This result demonstrates that the Protein Inference workflow can effectively identify single IL6 immunoprecipitated from human serum.

Sequencing Kit Data Sheet.indd 7 2/28/24 1:37 PM

FIGURE 5. INFERENCE SCORE OF IL6 AND THE SECOND INFERRED PROTEIN (ΔSCORE) FOR IL6 IMMUNOPRECIPITATED FROM HUMAN SERUM (2 LIBRARIES, EACH SEQUENCED 8 TIMES) AS ANALYZED WITH PROTEIN INFERENCE WORKFLOW.

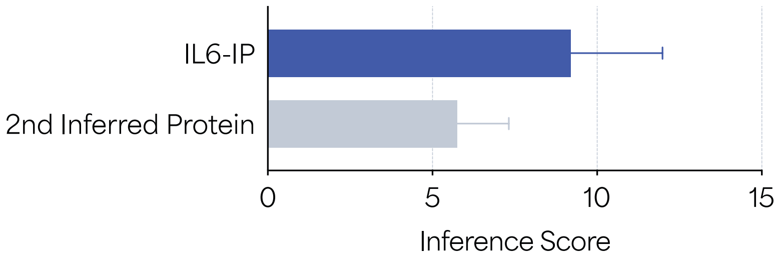

Another common application of interest is identifying unexpected or unknown bands in protein gels or other separation methods. In the example shown in Figure 6, purified CDNF was subjected to SDS-PAGE, resulting in two separate bands on the gel. Gel shifts may result from disulfide bonds altering the SDS binding capacity of proteins causing certain proteins to migrate more slowly through the gel. Both bands were excised, subjected to an in-gel digestion protocol for library preparation,4 and sequenced on Platinum. The results for both bands indicated that CDNF was inferred as the protein most likely present in the sample. For band A, CDNF had an Inference Score of 15.14, in contrast to 3.85 for the second inferred protein. For band B, CDNF had a score of 4.19, compared with 1.61 for the second protein. ΔScore of 11.29 (band A) and 2.58 (band B) corresponds to approximately an 80,000-fold (band A) and 14-fold (band B) difference in confidence levels between CDNF and the second inferred protein. The difference in ΔScore is likely attributed to a higher concentration of band A compared to band B, demonstrating the utility of protein inference for proteins of different concentrations. Future applications could include running protein extracts from serum or other biological samples on a gel, as well as excising bands and analyzing the unknown proteins using protein inference. In addition, protein inference can be useful when there are no antibodies available for the protein of interest. As we continue to develop and advance next-generation protein sequencing capabilities, we expect that inference using *de novo* (reference alignment free) residue calls will be possible with future enhancements.

FIGURE 6. SAMPLE RESULTS FROM PROTEIN INFERENCE ANALYSIS WORKFLOW ON UNKNOWN BANDS FROM SDS-PAGE GEL OF PURIFIED CDNF. INFERENCE SCORE AND ΔSCORE METRICS ARE NATURAL LOG CALCULATIONS OF THE FDR OF PROTEINS.

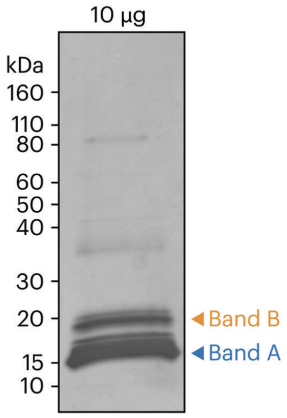

Band A

Protein	Rank	Inference Score (protein)
CDNF	1	15.14
HPSE2	2	3.85
UDB10	3	3.80
ZN211	4	3.36
HERP2	5	3.33

Band B

Protein	Rank	Inference Score (protein)
CDNF	1	4.19
ZN211	2	1.61
UDB10	3	1.39
LC7L3	4	1.39
CD1E	5	1.10

For Research Use Only. Not for use in diagnostic procedures.

Sequencing Kit Data Sheet.indd 8 2/28/24 1:37 PM

SUMMARY

Platinum and Sequencing Kit V2, with single-molecule and single-amino-acid resolution, enables new proteomic discoveries in an accessible and scalable benchtop solution. Through rigorous characterization across multiple sample types, we have demonstrated a 3-fold enhancement in amino acid recognition, low intra-chip variation for studies with two samples per run, and inference of single recombinant proteins, immunoprecipitated proteins, and protein bands from SDS-PAGE with the new Protein Inference workflow. The platform's use of a novel sequencing chip and N-terminal amino acid recognizers make protein identification and characterization with single amino acid molecule resolution possible, unlocking proteomics for every lab. For more information, visit www.quantum-si.com/products/.

PLATINUM SPECIFICATIONS

Instrument dimensions*	19.45 x 8.46 x 9.91 in<br>49.39 x 21.50 x 25.18 cm
Bench space	26.00 x 17.00 x 16.00 in<br>66.04 x 43.18 x 40.64 cm
Weight	27 lbs (12 kgs)
Power Cable (included)	IEC 60320 C13 connector<br>(IEC 60320 C14 inlet)
Fuses	250VAC 10A Fuse (2)
On-Board Storage	456 GB
Temperature	15-25°C<br>59-77°F
Humidity	35-55%

ORDERING INFORMATION

Product	Catalog Number
Platinum Instrument	910-10904-00
Sequencing Kit V2	910-00011-00

For Research Use Only. Not for use in diagnostic procedures.

Sequencing Kit Data Sheet.indd 9 2/28/24 1:37 PM

RELATED PRODUCTS

Product	Catalog Number
Library Preparation Kit - Lys-C	910-00012-00
Premium Service Contract	700-00005-00
Basic Service Contract	700-00006-00
Advanced Next-Generation Protein Sequencing™ Training	700-00004-00
Sequencing Control Peptide, SDQP155	910-00013-00

REFERENCES

1. Reed, B. D. et al. Real-time dynamic single-molecule protein sequencing on an integrated semi-conductor device. Science 378, 186–192 (2022).
2. Data Sheet: Platinum® Software Analysis Data Sheet.
3. App Note: Immunoprecipitation of IL-6 from Human Serum for Next-Generation Protein Sequencing on Platinum®.
4. Tech Note: Protein Identification Using Next-Generation Protein Sequencing™ of In-Gel Digested Proteins.

For Research Use Only. Not for use in diagnostic procedures.
