# Supplementary material for: Protein Sequencing with Single Amino Acid Resolution Discerns Peptides That Discriminate Tropomyosin Proteoforms
Source: J Proteome Res. 2025 Jun 10;24(8):3798–807. doi: 10.1021/acs.jproteome.4c00978 (PMC12322949; doi:10.1021/acs.jproteome.4c00978)
Supplement: Supplementary file 5 [file pr4c00978_si_005.pdf]

Platinum Analysis Software Data Sheet

Confidently Identify Proteins and Peptides with Single Amino Acid Resolution in a Simple User-friendly Analysis Workflow

February 2, 2024

INTRODUCTION

The Platinum instrument is the first next-generation protein sequencer™ that delivers single-molecule and single amino acid resolution in a user-friendly benchtop platform. Quantum-Si's Library Preparation Kits, Sequencing Kits, and Platinum Analysis Software provide everything you need to prepare, sequence, and analyze peptides and proteins on Platinum.

The Platinum Analysis Software is key for easily processing and viewing next-generation protein sequencing™ data generated by Platinum without the need for bioinformatics expertise. The generation of protein sequencing data begins with the use of Quantum-Si's Library Preparation Kits to digest and functionalize proteins into peptide libraries. Peptide libraries are then immobilized on sequencing chips, and a mixture of fluorescently labeled N-terminal amino acid (NAA) recognizers and aminopeptidases are added to the chip. Sequencing commences on the Platinum instrument as the recognizers bind to each NAA, creating a signal of fluorescent intensity and lifetime for each binding event. Aminopeptidases cleave each NAA exposing the next NAA for recognition, and the process repeats until the entire peptide is sequenced. Sequencing data is then transferred securely to the Platinum Analysis Software for processing and viewing results.

The Platinum Analysis Software is available through a secure user-friendly cloud environment enabling access to data from anywhere and supporting global collaborations. It provides users with an intuitive interface for setting up and planning runs. It consists of flexible workflow options for researchers who want to quickly identify peptides and infer proteins with confidence or for researchers who want to interrogate each amino acid to uncover new protein variants and modifications. Data generated can be exported for additional analysis and report generation.

INTUITIVELY SET-UP PROTEIN SEQUENCING RUNS AND FLEXIBLE ANALYSIS WORKFLOWS

The Platinum Analysis Software can be used to easily plan and set up protein sequencing runs. Researchers can create projects to organize related samples, libraries, and runs in one location. They have the option to run one library on a whole chip to maximize coverage or to split chips in half and run two libraries at a time to maximize throughput. Once the protein sequencing run plan is saved in the Platinum Analysis Software, researchers can select the saved run plan on the Platinum instrument to ensure protein sequencing data is automatically transferred and uploaded to the correct run record in the Platinum Analysis Software (Figure 1).

FIGURE 1: OVERVIEW OF PROTEIN SEQUENCING SET-UP USING PLATINUM ANALYSIS SOFTWARE

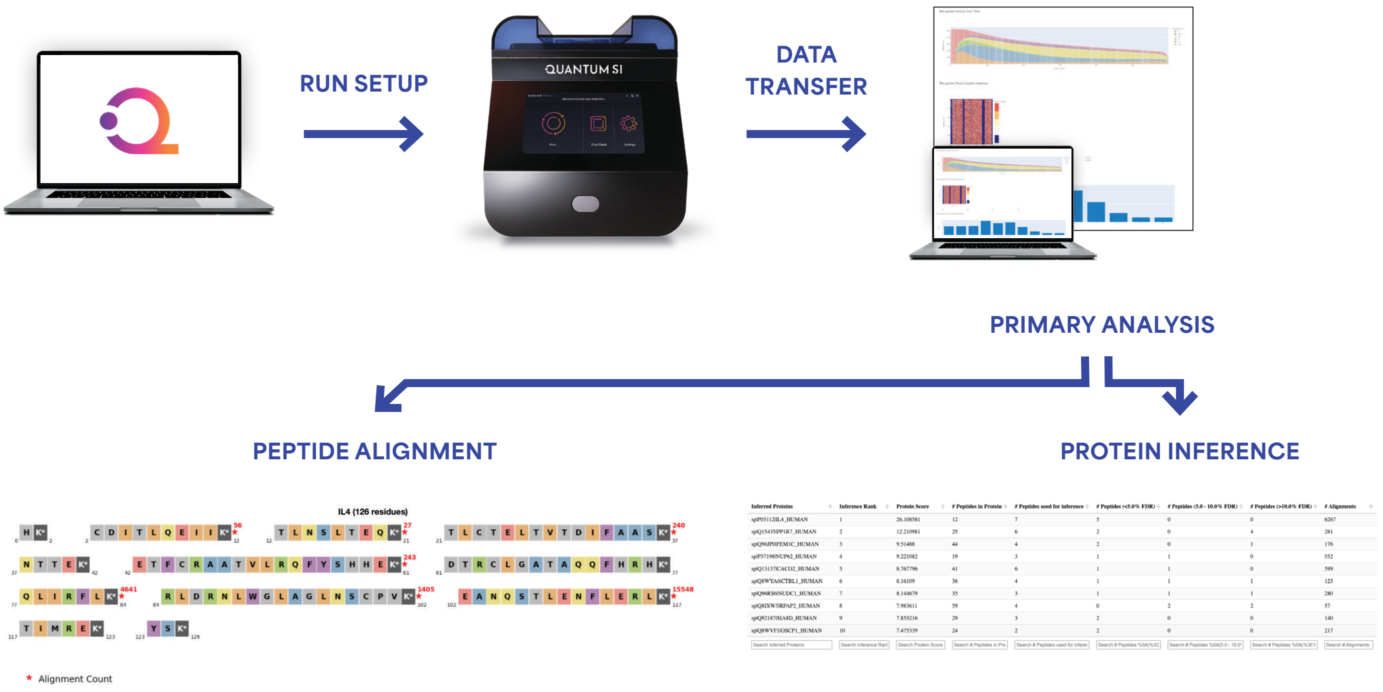

After run completion, the fluorescent signal, derived from the repeated on-off binding events of the recognizers and NAAs, is securely transferred to the Platinum Analysis Software. The Primary Analysis workflow is the first step in processing data which characterizes the apertures across the chip based on peptide loading, recognizer activity, recognizer reads, and recognizer read lengths (Figure 2). Primary analysis can be set-up during run creation for automatic processing or after the run has completed. Once the Primary Analysis data has been generated, researchers can choose to run Peptide Alignment or Protein Inference workflows on their data to interrogate the amino acid sequence, peptides identified, and proteins inferred.

For Research Use Only. Not for use in diagnostic procedures.

FIGURE 2: PRIMARY ANALYSIS CHARACTERIZES APERTURES BASED ON LOADING, RECOGNIZER ACTIVITY AND RECOGNIZER READ LENGTHS.

A) Loading Heatmap displays the spatial distribution of loaded wells in white and empty wells in blue across the Sequencing Chip. B) Recognizer Read Lengths Heatmap displays the distribution of reads based on length of recognizers with 4+ recognizers in red, 3 recognizers in orange, 2 recognizers in yellow, 1 recognizer in white, and 0 recognizers in blue. C) Recognizer Activity over Time displays the activity of recognizers over time. D) Recognizer Read Lengths Histogram displays the number of reads of each read length.

A. Loading Heatmap

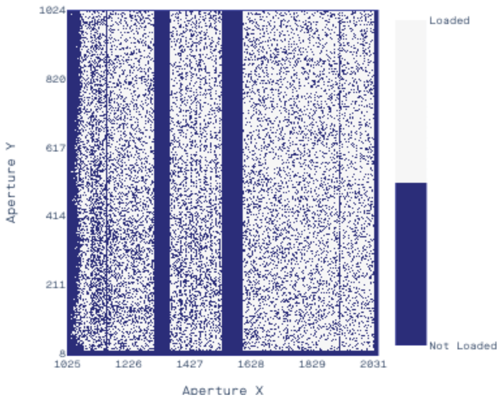

B. Recognizer Read Lengths Heatmap

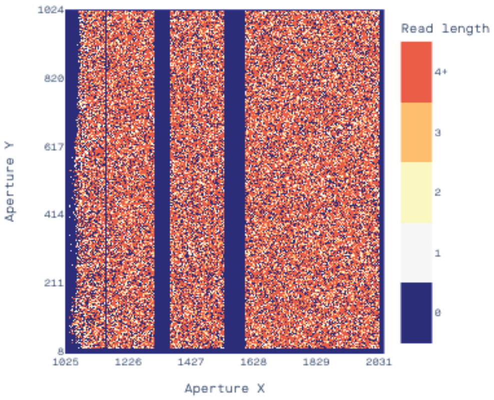

C. Recognizer Activity Over Time

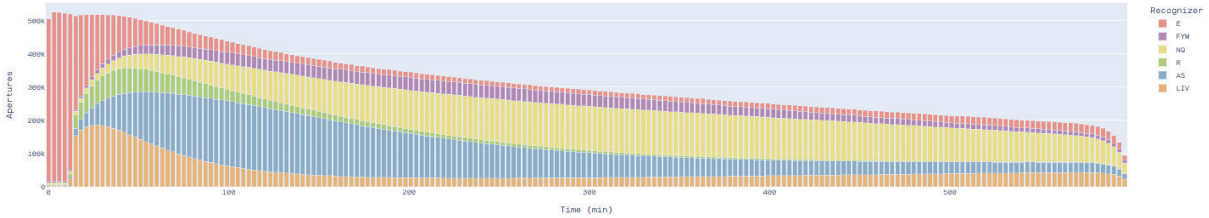

D. Recognizer Read Lengths Histogram

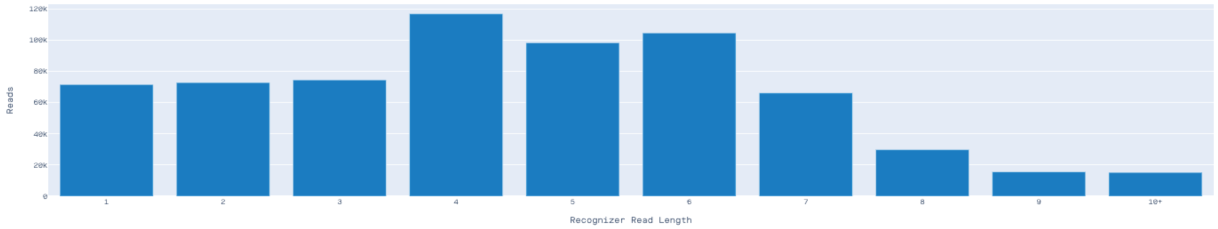

For Research Use Only. Not for use in diagnostic procedures.

CONFIDENTLY INTERROGATE AMINO ACID AND PEPTIDE SEQUENCE OF PROTEINS

The Peptide Alignment workflow is ideal for analyzing peptide or protein samples with a known or expected identity. For example, peptide barcodes, enriched peptides, immunoprecipitated proteins, or known recombinant proteins or antibodies would all benefit from sequence analysis using the Peptide Alignment workflow.

Researchers provide a FASTA file for a known or expected protein or peptide sequence to the Peptide Alignment workflow. The workflow then performs an in silico digest of the protein based on the library preparation kit used (e.g. Lys-C digest with the Library Preparation Kit - Lys-C) and generates a predicted profile to be used as a reference for alignment. Reads are then aligned to the reference profile based on the correspondence of observed recognition segments to the expected reference profile, using recognizer identity.

The data is reported as a complete protein sequence for easy results interpretation with all the possible recognized amino acid residues shaded in color and the number of alignments for each peptide indicated in red with a star (Figure 3a). Furthermore, a false discovery rate (FDR) is assigned to each peptide using a decoy generation that was adapted from methods used in peptide identification by mass spectrometry. Peptides with a FDR of less than 0.05 are displayed in green, a FDR of 0.05 or more but less than 0.10 are displayed in orange, and a FDR of greater than or equal to 0.10 are displayed in red (Figure 3b).

FIGURE 3: PEPTIDE ALIGNMENT AND FALSE DISCOVERY RATE PROFILES

A) The complete protein sequence is displayed with identified peptides indicated in red. B) Peptide alignments and false discovery rate (FDR) are displayed for each peptide.

A. Protein Sequence

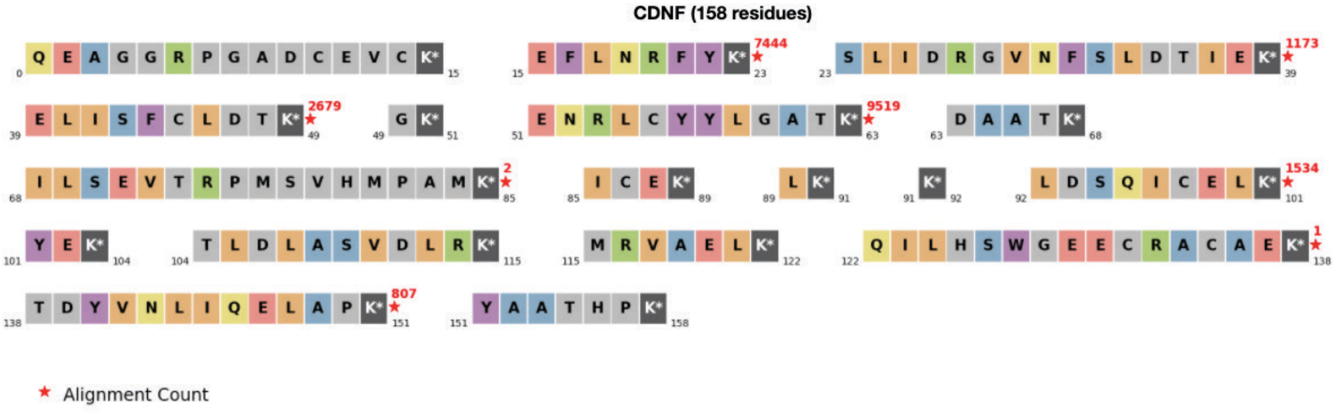

For Research Use Only. Not for use in diagnostic procedures.

B. Peptide Alignments

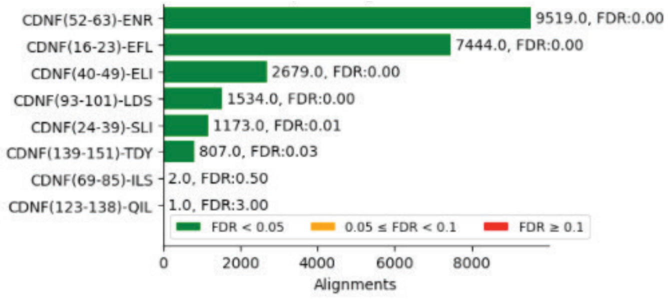

Data can be viewed as a summary coverage plot indicating the coverage for each amino acid residue and the average pulse duration and interpulse duration for each amino acid residue. The Recognition Segment (RS) represents the contiguous time segment of a trace containing sustained pulsing that is self-consistent in the properties of its pulses which are used to align to the amino acid sequence. The RS start time and duration are also displayed for each segment (Figure 4a). To explore each peptide sequence more deeply, the individual profiles that make up the total alignments and coverage plots can be visualized and accessed in output data files (Figure 4b).

FIGURE 4: RECOGNITION PROFILES MAKE UP PEPTIDE ALIGNMENTS

A) The coverage plot for each peptide summarizes the properties of each recognition segment (RS) that is aligned to the amino acid sequence. B.) The individual profiles that make up the alignments for each peptide are provided for deeper exploration.

A.

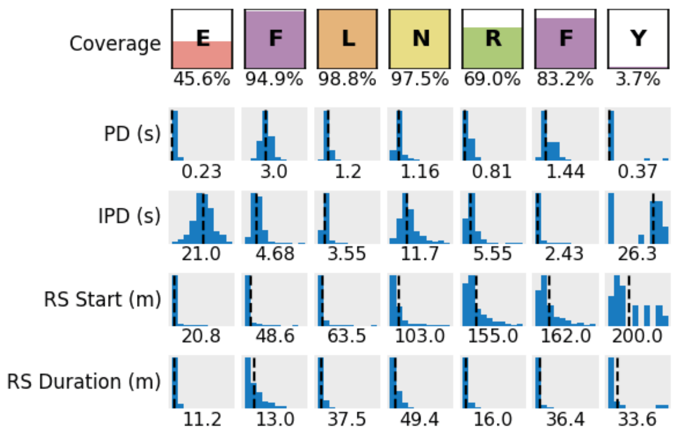

B.

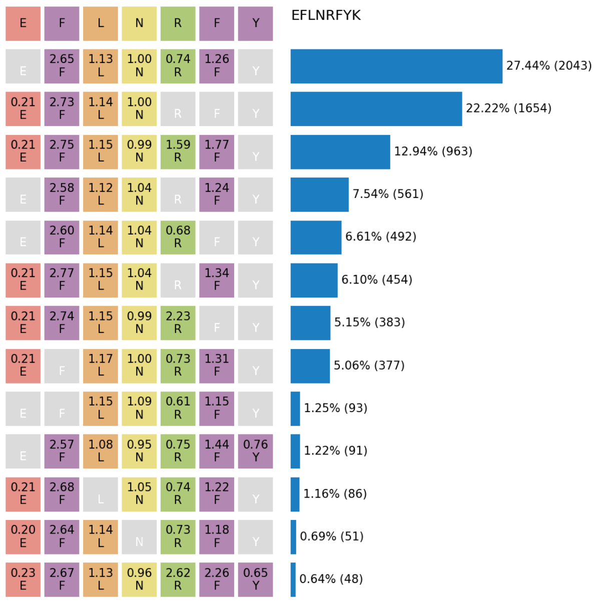

For Research Use Only. Not for use in diagnostic procedures.

EFFICIENTLY INFER PROTEINS FROM PANELS OF THOUSANDS OF PROTEINS

The Protein Inference workflow is designed for researchers who want to investigate protein samples that may have unknown proteins. For example, co-immunoprecipitated proteins and protein mixtures can be sequenced using Platinum, and proteins within the sample can be inferred using the Protein Inference workflow.

The Protein Inference workflow uses more rigorous criteria for protein inference and ranking compared to the Peptide Alignment workflow to ensure the utmost confidence in the results that are displayed. Researchers have two options when selecting a reference sequence for protein inference. The first option is a pre-defined panel of human proteins developed and provided by Quantum-Si which contains 7,921 proteins that have a molecular weight between 10 kDa and 70 kDa. All of the selected proteins contain at least three in silico lysine digested peptides with at least three unique and visible residues. The pre-defined panel contains 168,139 peptides with 1,108,935 visible residues. The pre-defined panel is recommended for analyzing human protein samples that might contain unknown proteins. The second option is to upload user-defined reference sequence as they would do with the Peptide Alignment workflow. Researchers have the option to select multiple references as an input for protein inference.

Inferred proteins are displayed as a table by ranking the proteins with the highest Protein Score. The Protein Score is calculated by aggregating the false discovery rate of all the in silico digested peptides generated from the protein sequence. The table also displays the number of peptides within the protein and the number of peptides that were used for inference. Further confidence in the data is provided by displaying the number of peptides by a %FDR which is calculated using a decoy set of peptides, and the total number of alignments (Figure 5). In addition to the summary report of inferred proteins, the FDR and number of alignments for each peptide is provided in a table format for quick interpretation of the results (Figure 6). The searchable interface enables the user to sift through hundreds to thousands of potential proteins for deep investigation.

FIGURE 5: RANK ORDER OF INFERRED PROTEINS

<table>
<tr><th>Inferred Proteins</th><th>Inference Rank</th><th>Protein Score</th><th># Peptides in Protein</th><th># Peptides used for inference</th><th># Peptides (<5.0% FDR)</th><th># Peptides (5.0 - 10.0% FDR)</th><th># Peptides (>10.0% FDR)</th><th># Alignments</th></tr>
<tr><td>sp|P05112|IL4_HUMAN</td><td>1</td><td>26.108581</td><td>12</td><td>7</td><td>5</td><td>0</td><td>0</td><td>6267</td></tr>
<tr><td>sp|Q15435|PP1R7_HUMAN</td><td>2</td><td>12.210981</td><td>25</td><td>6</td><td>2</td><td>0</td><td>4</td><td>281</td></tr>
<tr><td>sp|Q96JP0|FEM1C_HUMAN</td><td>3</td><td>9.51488</td><td>44</td><td>4</td><td>2</td><td>0</td><td>1</td><td>176</td></tr>
<tr><td>sp|P37198|NUP62_HUMAN</td><td>4</td><td>9.221082</td><td>19</td><td>3</td><td>1</td><td>1</td><td>0</td><td>552</td></tr>
<tr><td>sp|Q13137|CACO2_HUMAN</td><td>5</td><td>8.767796</td><td>41</td><td>6</td><td>1</td><td>1</td><td>0</td><td>599</td></tr>
<tr><td>sp|Q8WYA6|CTBL1_HUMAN</td><td>6</td><td>8.16109</td><td>36</td><td>4</td><td>1</td><td>1</td><td>1</td><td>123</td></tr>
<tr><td>sp|Q96RS6|NUDC1_HUMAN</td><td>7</td><td>8.144679</td><td>35</td><td>3</td><td>1</td><td>1</td><td>1</td><td>280</td></tr>
<tr><td>sp|Q8IXW5|RPAP2_HUMAN</td><td>8</td><td>7.983611</td><td>59</td><td>4</td><td>0</td><td>2</td><td>2</td><td>57</td></tr>
<tr><td>sp|Q92187|SIA8D_HUMAN</td><td>9</td><td>7.853216</td><td>29</td><td>3</td><td>2</td><td>0</td><td>0</td><td>140</td></tr>
<tr><td>sp|Q8WVF1|OSCP1_HUMAN</td><td>10</td><td>7.475339</td><td>24</td><td>2</td><td>2</td><td>0</td><td>0</td><td>217</td></tr>
<tr><td>Search Inferred Proteins</td><td>Search Inference Ranl</td><td>Search Protein Score</td><td>Search # Peptides in Pro</td><td>Search # Peptides used for inferer</td><td>Search # Peptides %0A(%3C</td><td>Search # Peptides %0A(5.0 - 10.0</td><td>Search # Peptides %0A(%3E1</td><td>Search # Alignments</td></tr>
</table>

For Research Use Only. Not for use in diagnostic procedures.

FIGURE 6: PEPTIDE ALIGNMENTS AND FALSE DISCOVERY RATE FROM INFERRED PROTEINS

<table>
<tr><th>Protein Inferred</th><th>Peptide</th><th>Length</th><th>FDR</th><th># Alignments</th></tr>
<tr><td>sp|P05112|IL4_HUMAN</td><td>sp|P05112|IL4_HUMAN(127-141)-EAN</td><td>15</td><td>0.000236</td><td>4237</td></tr>
<tr><td>sp|P05112|IL4_HUMAN</td><td>sp|P05112|IL4_HUMAN(102-108)-QLI</td><td>7</td><td>0.000517</td><td>1936</td></tr>
<tr><td>sp|P05112|IL4_HUMAN</td><td>sp|P05112|IL4_HUMAN(109-126)-RLD</td><td>18</td><td>0.025641</td><td>39</td></tr>
<tr><td>sp|P05112|IL4_HUMAN</td><td>sp|P05112|IL4_HUMAN(46-61)-TLC</td><td>16</td><td>0.032258</td><td>31</td></tr>
<tr><td>sp|P05112|IL4_HUMAN</td><td>sp|P05112|IL4_HUMAN(67-85)-ETF</td><td>19</td><td>0.045455</td><td>22</td></tr>
<tr><td>sp|Q9UIL4|KIF25_HUMAN</td><td>sp|Q9UIL4|KIF25_HUMAN(118-136)-VEV</td><td>19</td><td>0.25</td><td>4</td></tr>
<tr><td>sp|P05112|IL4_HUMAN</td><td>sp|P05112|IL4_HUMAN(27-36)-CDI</td><td>10</td><td>NaN</td><td>1</td></tr>
<tr><td>sp|P05112|IL4_HUMAN</td><td>sp|P05112|IL4_HUMAN(37-45)-TLN</td><td>9</td><td>NaN</td><td>1</td></tr>
<tr><td>IL4</td><td>Search Peptide</td><td>Search Length</td><td>Search FDR</td><td>Search # Alignments</td></tr>
</table>

EASILY DOWNLOAD RAW DATA FOR ADDITIONAL PROCESSING

The workflows provided in the Platinum Analysis Software are designed to enable easy interpretation of protein sequences without the need for bioinformatics expertise. However, researchers may want to process the raw data files with additional analysis for different applications.

Files from the Primary Analysis, Peptide Alignment, and Protein Inference workflows are all available for download. The Primary Analysis files include the filtered recognition segments from all apertures that contain reads with a recognizer read length of 4 or more and at least 3 unique recognizers, including the recognition segment start and end time, the pulse duration, and the interpulse duration data. The Peptide Alignment files contain alignment results from reads that mapped to the user provided reference sequence and met a minimum alignment score, including the peptide, recognition segment, reference index, and alignment score for each aperture. The Protein Inference workflow exports files with the information displayed in Figures 5 and 6.

SUMMARY

Platinum Analysis Software contains a complete end-to-end solution for setting up and processing next-generation protein sequencing data on Platinum. Users can easily set up runs with an intuitive workflow, data is automatically transferred to a secure cloud interface, and researchers have the option to analyze amino acids, peptides, and proteins with flexible workflows.

Platinum Analysis Software is included with the purchase of every Platinum instrument and cloud data storage is unlimited for the first year and with Premium Service Contracts. For more information, visit www.quantum-si.com/products/.

ORDERING INFORMATION

Product	Catalog Number
Platinum Instrument	910-10904-00
Premium Service Contract	700-00005-00

RELATED PRODUCTS

Product	Catalog Number
Library Preparation Kit - Lys-C	910-00012-00
Sequencing Kit V2	910-00011-00
Basic Service Contract	700-00006-00
Advanced Next-Generation Protein Sequencing™ Training	700-00004-00
Sequencing Control Peptide, SDQP155	910-00013-00

For Research Use Only. Not for use in diagnostic procedures.
